# Acidity‐Aided Surface Modification Strategy to Enhance In Situ MnO_2_ Deposition for High Performance Zn‐MnO_2_ Battery Prototypes

**DOI:** 10.1002/smll.202311933

**Published:** 2024-03-28

**Authors:** Manas Ranjan Panda, Sally El Meragawi, Meysam Sharifzadeh Mirshekarloo, Wanqing Chen, Mahdokht Shaibani, Mainak Majumder

**Affiliations:** ^1^ Nanoscale Science and Engineering Laboratory (NSEL) Department of Mechanical and Aerospace Engineering Monash University Clayton VIC 3800 Australia; ^2^ ARC Research Hub for Advanced Manufacturing with 2D materials (AM2D) Monash University Clayton VIC 3800 Australia; ^3^ Department of Chemical and Environmental Engineering, RMIT University Melbourne VIC 3001 Australia

**Keywords:** cell architecture, current collectors, electrolyte engineering, high voltage Zn–MnO_2_ aqueous battery

## Abstract

Zn–MnO_2_ batteries offer cost‐effective, eco‐friendly, and efficient solutions for large‐scale energy storage applications. However, challenges, like irreversible cathode reactions, prolonged cyclability, and electrolyte stability during high‐voltage operations limit their broader application. This study provides insight into the charge–discharge process through in situ deposition of active *β*‐MnO_2_ nanoflakes on a carbon‐based current collector. The study elucidates the effect of pH and electrolyte concentration on chemical conversion reactions with Zn, in particular focus on their impact on the two‐electron MnO_2_/Mn^2+^ reaction crucial for high voltage operation. The electrolyte, characterized by being relatively lean in Mn^2+^ and with a targeted low pH, enables extended cycling. This research achieves greater cycling durability by integrating a carbon‐based cathode current collector with high density of structural defects in combination with cell architectures suitable for large‐scale energy storage. A flooded stack‐type Zn–MnO_2_ battery prototype employing the optimized electrolyte demonstrates a high discharge voltage (≈2 V) at a substantial discharge current rate of 10 mA cm^−2^. The battery exhibits an impressive areal capacity of ≈2 mAh cm^−2^, maintaining ≈100% capacity retention over 400 cycles. This research establishes a promising practical, and cost‐effective cathode‐free design for Zn–MnO_2_ batteries, that minimizes additional processing and assembly costs.

## Graphical abstract

This study optimizes electrolyte pH to extend cycling by influencing two‐electron MnO_2_/Mn^2+^ reaction for high voltage operation. It integrates a carbon‐based current collector with a high density of structural defects for enhanced cycling. With impressive performance and minimal decay over 400 cycles in a flooded stack‐type cell architecture, these batteries present a promising practical solution for large‐scale energy storage applications.

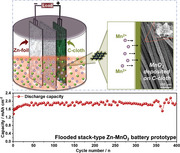

## 1 Introduction

Li‐ion batteries (LIBs), recognized as the foremost battery chemistry, offer advantages such as high energy density, extended cycle life, and high operating voltage. However, their widespread adoption in large‐scale stationary applications faces challenges related to limited materials supply, concerns about flammability, and associated environmental and safety issues.^[^ ^1^, ^2^ ^]^ On the other hand, aqueous batteries present an alternative as low‐cost systems with proven potential for scalability in stationary applications. They offer additional advantages, including enhanced safety features and the use of sustainable materials.^[^ ^2^, ^3^, ^4^ ^]^ Amongst the aqueous batteries, lead‐acid batteries (LABs) dominate the industry, accounting for more than half of the global battery market. Despite this, the use of lead has caused significant environmental concerns and the battery performance of LABs is marked by low energy density (≈30–50 Wh kg^−1^), limited cycle life, and slow, inefficient charging.^[^ ^5^ ^]^ A low‐cost, environmentally benign aqueous battery substitute to the existing LABs is eminently necessary for the current market.^[^ ^5^, ^6^, ^7^, ^8^ ^]^

Zinc metal stands out as a prime anode option for aqueous batteries, attracting attention because of its high theoretical capacity (820 mAh g^−1^), which rivals that of LIBs.^[^ ^9^, ^10^, ^11^ ^]^ Cathode candidates, including oxides, phosphates, sulfides, oxynitrides, and MXenes, have exceptional performance in LIBs that is not achieved in their Zn analog due to limitations arising from intrinsic properties associated with the large Zn^2+^ ions.^[^ ^12^ ^]^ Among oxides, manganese and vanadium‐based materials have emerged as the most promising practical cathode contenders, with particular emphasis given to their specific energy and cost‐effectiveness.^[^ ^13^ ^]^ Manganese oxides (MnO_2_) are notably superior due to its low‐cost abundant reserves and in combination with non‐flammable, low‐toxicity electrolytes, is preferable as a cathode that can augment safety concerns and reduce manufacturing costs below $10 kWh^−1^.^[^ ^14^, ^15^ ^]^ Recent advancements in Zn–MnO_2_ batteries focus on improving energy density, extending the voltage window, safety, and environmental sustainability through the examination of various cathode materials, electrolyte engineering, and anode modification.^[^ ^11^ ^]^ Progress has also been made in zinc anode materials, electrolyte formulations, and cell designs, leading to enhanced stability and efficiency.^[^ ^11^, ^16^ ^]^ However, challenges persist that contribute to limited rate capability, elevated cell voltage, and MnO_2_ structural degradation during cycling that hinder long‐term reliability. Moreover, optimizing scalability and cost‐effectiveness for widespread commercial use remains a significant hurdle. Addressing these challenges is imperative for realizing the full potential of Zn–MnO_2_ batteries in practical applications within the energy storage landscape. Ongoing research efforts are crucial in overcoming these obstacles and advancing the utilization of Zn–MnO_2_ batteries in various sectors.

Commercially available rechargeable Zn–MnO_2_ batteries utilize alkaline KOH as the electrolyte and as such, suffer from a lower voltage and energy density derived from the reliance on a single‐electron redox mechanism.^[^ ^10^ ^]^ Recently, Zn–MnO_2_ batteries have shown increased capacity by invoking two‐electron (e^−^) reactions.^[^ ^9^, ^10^, ^14^, ^17^, ^18^, ^19^, ^20^, ^21^ ^]^ In general, the reduction of MnO_2_ contributes to 308 mAh g^−1^ of the theoretical specific capacity per each e^−^ involved.^[^ ^8^, ^18^ ^]^ However, the reliable involvement of 2nd e^−^ reduction chemistry is greatly diminished by the formation of inactive, resistive compounds.^[^ ^14^, ^18^ ^]^ The one‐electron mechanism is characterized by the formation of several electrochemically inactive intermediates including tunnel‐type Zn*_x_* Mn_2_O_4_, spinel ZnMn_2_O_4_, and other relevant complex composites.^[^ ^10^, ^17^, ^18^, ^19^, ^20^, ^21^ ^]^ The formation of these oxides contributes to lower energy density and capacity fading, particularly during deep discharge.^[^ ^22^ ^]^ The irreversible formation of Mn_3_O_4_ prevents the electrochemically active MnO_2_ from further participating in redox reactions which leads to low Coulombic efficiency (CE),^[^ ^23^ ^]^ and energy density. The lack of a second electron discharge plateau in Zn–MnO_2_ is a clear indicator of Zn(OH)_4_^2−^ and the subsequent formation of ZnMn_2_O_4_ leads to a loss in conductivity, electrode pore closures, and lower voltages, reducing the second electron energy of MnO_2_.^[^ ^11^, ^16^ ^]^

Achieving a high voltage window and long‐term cycling stability hinges on the stability of the aqueous electrolyte, which in turn is also influenced by the type of products being formed during battery cycling.^[^ ^10^ ^]^ Literature has demonstrated the feasibility of improving the voltage window of the Zn–MnO_2_ battery by altering the pH of the electrolyte. Substituting standard hydroxide solutions (KOH, NaOH, and LiOH)^[^ ^23^, ^24^, ^25^, ^26^, ^27^ ^]^ with ZnSO_4_ and MnSO_4_ salts^[^ ^28^, ^29^, ^30^, ^31^, ^32^, ^33^ ^]^ or introducing H_2_SO_4_ can invoke the two‐electron mechanism,^[^ ^14^, ^34^ ^]^ particularly in high‐voltage operations. The Pourbaix diagram^[^ ^35^ ^]^ for manganese oxide compounds reveals that, at high pH, MnO_2_ redox occurs at a low potential of 0.250 V versus SHE, and favors transformation to undesirable Mn_3_O_4_. However, lowering the pH into the acidic range facilitates the reduction of Mn^4+^ ions to Mn^2+^, preventing the formation of Mn_3_O_4_ and significantly improving the cathode's reaction potential to above 1.2 V versus SHE. While an acidic electrolyte enhances the cathode's reaction potential, it also increases the reaction potential of the Zn anode. In an alkaline pH, the Zn anode potential is −1.199 V versus SHE, which shifts to −0.762 V versus SHE in acidic media, lowering the overall cell potential.^[^ ^36^ ^]^ Zn–MnO_2_ batteries utilizing acidic gelled electrolytes operate at cell voltages as high as 2.8 V as demonstrated by Yadav et. al.^[^ ^37^ ^]^ Research by Pan et al. has shown that the use of mildly acidic electrolytes in aqueous Zn–MnO_2_ batteries improves battery cyclability and capacity retention.^[^ ^38^ ^]^ However, acidic electrolytes also induce a corrosion reaction at the Zn anode, leading to challenges such as Zn dissociation, H_2_ release, decreased capacity, and eventual cell failure.^[^ ^39^, ^40^ ^]^

It is imperative to investigate the microstructure and morphology of MnO_2_ deposited on various types of carbon sources used as current collectors (CCs), as it plays a crucial role in cycling stability and ensuring a structure that facilitates ion transport.^[^ ^41^ ^]^ MnO_2_ can manifest in several crystallographic forms (*α*‐MnO_2_, *β*‐MnO_2_, *γ*‐MnO_2_, and *δ*‐MnO_2_) where the unit structure of MnO_6_ octahedra are connected in various ways to form a rich polymorphism that has a profound influence on how Zn^2+^ ions are stored at the edges/corners of the MnO_6_ octahedra units.^[^ ^9^, ^14^, ^42^ ^]^ Among these, *α*‐MnO_2_ and *β*‐MnO_2_ have unique microstructures and morphologies suitable to providing decent capacity and are widely explored as electrode candidates for aqueous Zn–MnO_2_ batteries.^[^ ^13^, ^19^, ^43^, ^44^ ^]^ You et al. performed a comparative study on various forms of MnO_2_ (*α*, *β*, and *δ*‐MnO_2_) and concluded that *β*‐MnO_2_ possesses better thermodynamic stability and a more regular morphological structure.^[^ ^41^ ^]^ However, within the realm of the two‐electron mechanism, the behaviors of the polymorphs have not been extensively explored in the literature including aspects such as the structural stability under battery cycling conditions.

A key advantage of the battery chemistry presented in this work is its seemingly straightforward configuration, requiring only a zinc metal anode, an aqueous electrolyte composed of Zn and Mn salts, and a platform for MnO_2_ deposition in the form of a carbon CC with a structure appropriate for fast Mn^2+^ transport. This simplicity allows for a diverse range of choices in material selection and device configuration. At the heart of this device lies a simple process of in situ deposition of *β*‐MnO_2_ particles during charging which becomes the active material in the battery. A comprehensive understanding of this phenomenon and its correlation with performance is essential for the development of optimal prototype devices. In this study, we elucidate the influence of the physicochemical properties of the CC, discuss the criteria for the judicious selection of electrolytes to enable the two‐electron MnO_2_/Mn^2+^ reaction mechanism in assembled devices, and provide our perspective on the fabrication of efficient rechargeable Zn–MnO_2_ battery prototypes.

## 2 Results and Discussion

### 2.1 Influence of Carbon Current Collector on the Performance of Zn–MnO_2_ Batteries

**Figure 1:**
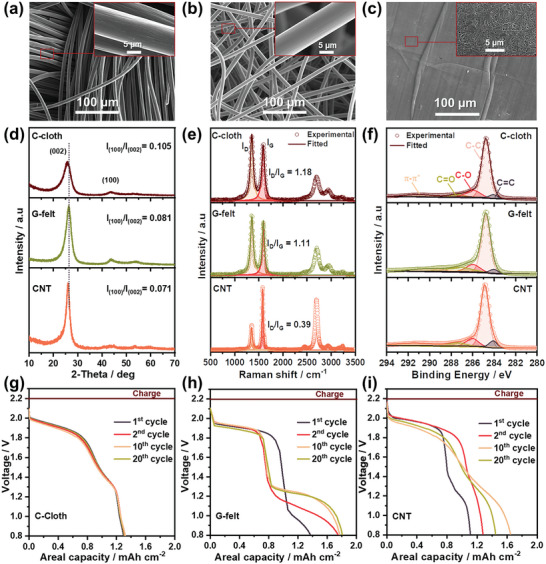
Correlation between the structure of the carbon‐based CCs and the cycling electrochemical reactions. a–c) FE‐SEM images showing the surface textures and the corresponding high‐resolution images (inset) of C‐cloth, G‐felt, and CNT d–f) XRD, Raman, and X‐ray photoelectron spectroscopy (XPS) spectra of C‐cloth, G‐felt, and CNT illustrate the structural and chemical differences between carbon sources. g–i) The variation in the discharge curves, cell voltage, and areal capacities of Zn–MnO_2_ batteries achieved by implementing different carbon sources as cathode CCs at a discharge current rate of 6 mA cm^−2^.

It is desirable to enhance either the specific capacity or the working area of electrodes with CCs possessing high available surface area for MnO_2_ deposition and good ion conductivity.^[^ ^9^ ^]^ To understand the interplay of properties such as an accessible surface area for active site deposition, structural defects, disorder, crystallinity, and the presence of oxygen‐containing functional groups on the long‐term stability and high capacity performance of Zn–MnO_2_ batteries, this study explored distinct carbon sources that include Carbon cloth, Graphite felt, and Carbon nanotubes (C‐cloth, G‐felt, and CNT) as CCs. **Figure** **1**a–c illustrates the difference in surface textures of C‐cloth, G‐felt, and CNT where the Field Emission Scanning Electron Microscope (FE‐SEM) images of C‐cloth and G‐felt have shown high available void and channels for electrolyte transport. In comparison, CNT has a dense surface with limited channels available for the transport of electrolyte (Mn^2+^ ions) into the structure of the host material and may lead to the blockage of the void space and enhance capacitance fading. Figure 1d shows the X‐Ray diffraction (XRD) patterns of pristine C‐cloth, G‐felt, and CNT CCs, the major reflection XRD peaks, at ≈26° and ≈43°, correspond to the reflections of the (002) and (100) crystallographic planes, which is representative of crystalline components with several orders of basal plane alignment.^[^ ^45^, ^46^, ^47^ ^]^ The (002) peak intensity of CNT CC has higher order compared to G‐felt and C‐cloth CCs, confirming the higher graphitic order of the carbon lattice.^[^ ^47^ ^]^

Further, the position of the (002) peak at a lower 2*θ* value of C‐cloth (25.78°) relative to both G‐felt (25.92°) and CNT CCs (26.05°) indicates an expansion to a higher lattice parameter for C‐cloth CC that is better suited for the deposition of MnO_2_ during charging. The ratio I_(100)_/I_(002)_, which can be taken as an indicator of the in‐plane crystallinity was observed to decrease from 0.105 in C‐cloth to 0.071 in CNT CC, which indicates the presence of more defects on C‐cloth surfaces and the partial disordering of in‐plane reflection planes.^[^ ^46^, ^47^ ^]^ Additionally, Raman spectra of C‐cloth, G‐felt, and CNT CCs were performed, and the intensity ratio of the D and G‐bands (I_D_/I_G_) were obtained to investigate the order of crystallinity. A lower I_D_/I_G_ reflects a higher degree of order in the crystal structure, the degree of p‐conjugation, and a lower number of defects.^[^ ^48^, ^49^, ^50^, ^51^, ^52^ ^]^ The I_D_/I_G_ ratio for the C‐cloth, G‐felt, and CNT samples were found to be 1.18, 1.11, and 0.39, respectively, as determined by the deconvolution of the corresponding I_D_ and I_G_ peaks shown in Figure 1e. C‐sources with different I_D_/I_G_ ratios have different electronic interactions with the Mn^2+^ ion, which can facilitate or hinder electron transport between the C‐sources and Mn^2+^ during MnO_2_ deposition (observed in the change in the first cycle discharge curves in Figure 1g; Figure S1, Supporting Information). The C‐cloth CC has a higher I_D_/I_G_ ratio (1.18) due to the increasing disorder of the host structure and higher number of defects, which enhances the adsorption of Mn^2+^ ions into the host structure. Similarly, the lower I_D_/I_G_ ratio for the G‐felt CC (1.11) and CNT CC (0.39) samples align with a host structure that has an inherent ion transport resistance leading to the lower adsorption of Mn^2+^. Further, the second‐order Raman region (2000 to 3000 cm^−1^) shows two distinct peaks for all carbon CCs. The CNT sample has a higher peak intensity due to the higher degree of graphitization, where the higher wavenumber peaks disappeared (Figure 1e).^[^ ^48^, ^49^, ^50^ ^]^ Figure 1f shows the C 1s XPS spectra of C‐cloth, G‐felt and CNT samples, and the corresponding bonding content ratios are listed in Table S1 (Supporting Information). Figure S2a (Supporting Information) shows the wide scan survey XPS and O 1s spectra confirming the presence of C and O elements in the C‐cloth, G‐felt, and CNT CCs samples. The C 1s XPS spectra with deconvoluted peaks situated at 284.07, 284.03 and 284.08 eV for (C═C), 284.76 eV, 284.77, and 284.88 eV for (C─C), 285.94, 285.96 and 286.08 eV for (C─O), and 287.48, 287.16 and 286.98 eV for (O─C═O) for C‐cloth, G‐felt, and CNT CC samples, respectively (Figure 1f).^[^ ^9^, ^53^, ^54^, ^55^, ^56^, ^57^ ^]^ The C‐cloth CC sample showed high C─O content compared to the CNT CC sample, further confirming that the percentage of oxygen‐containing functional groups is higher in C‐cloth and G‐felt CCs and may be the source of the high defect count observed in Raman and thus represent a higher number of active sites for the robust deposition of Mn^2+^ on the surface of C‐cloth and G‐felt CCs.^[^ ^9^, ^53^, ^54^, ^55^, ^56^, ^57^, ^58^, ^59^, ^60^ ^]^

Figure 1g–i illustrates the outcomes of chronoamperometric (CA) charging of Zn–MnO_2_ cells at a constant potential of 2.2 V and discharge curves at an applied constant current of 6.0 mA cm^−2^. The discharge plateau and areal capacities for three distinct types of CCs are depicted in Figure 1g–i. In particular, Figure 1g illustrates the C‐cloth's discharge potential plateau ≈1.8–2 V versus Zn^2+^/Zn, with a consistent areal capacity over the initial 20 cycles. However, for the CNT CC (Figure 1i), a rapid decline in areal capacity is observed under the same charging voltage and current conditions. Although G‐felt initially displays a stable areal capacity, its high‐voltage plateau shortens after the first cycle (Figure 1h), indicating unsuitability for operations at high voltages. The observed lower order crystallinity, high available void and channels for electrolyte transport, and the more defective structure of C‐cloth are identified as factors responsible for the robust MnO_2_ deposition during the Zn–MnO_2_ battery charging process. Consequently, due to its appropriate cycling stability, ion transport facilitation, and overall performance, C‐cloth has been selected as the preferred CC for further testing.

### 2.2 The Nature of Electrodeposited MnO_2_ on C‐cloth Current Collector

**Figure 2:**
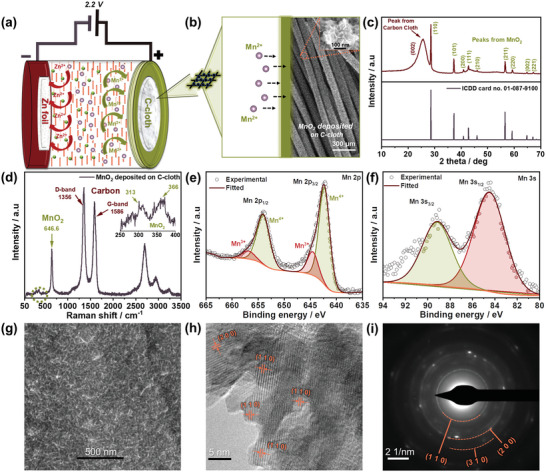
A schematic illustration of the in situ synthesis of *β*‐MnO_2_ active material, crystal structure, and microstructural analysis, and electron microscopy studies of the as‐deposited MnO_2_ on the C‐cloth CC after the first full charge. a) Schematic of the in situ synthesis of MnO_2_ and the Zn–MnO_2_ batteries configuration in a 1 m MnSO_4_ and 1 m ZnSO_4_ electrolyte system. b) The electrodeposition process for the formation of MnO_2_ active material during the charging process. c) XRD pattern; d) Raman spectra; e,f) HR‐XPS spectra of Mn 2p and Mn 3s of MnO_2_ deposited on C‐cloth after full charge. g) FE‐SEM image showing the uniform deposition of MnO_2_ on the surface of the C‐cloth CC. h,i) The HR‐TEM images showing the lattice fringes and the SAED pattern for deposited MnO_2_ nanoparticles.

The nature of the electrodeposited MnO_2_ microstructure on C‐cloth is crucial for elucidating the behavior of the two‐electron mechanism and understanding structural and cycling stability and ion transport during battery cycling. The Zn–MnO_2_ battery was charged at a constant voltage of 2.2 V versus Zn^2+^/Zn, and discharged at a constant current typically employed for Zn–MnO_2_ cells,^[^ ^9^, ^14^ ^]^ as shown in **Figure** **2**a. The MnO_2_ active material is deposited on the surface of the CC in the first cycle of battery operation (Figure 2a,b). To confirm the nature of the active Mn oxide phase, XRD, Raman, and Transmission electron microscopy (TEM) studies were used to characterize the as deposited CC after the first full charge. The XRD pattern confirms the deposition of *β*‐MnO_2_ (ICDD card number 01‐087‐9100) as shown in Figure 2c.^[^ ^42^, ^45^ ^]^ All the peaks are indexed with the planes corresponding to the *β*‐MnO_2_ phase, and exhibit high crystallinity as observed from the intensity of the (110) plane of *β*‐MnO_2_, whereas the broad peak ≈25° corresponds to the peak from C‐cloth CC.^[^ ^42^, ^45^ ^]^

Raman spectroscopy was carried out after full charge as shown in Figure 2d. The two intense peaks at 1356 and 1586 cm^−1^ correspond to the D and G bands originating from the C‐cloth CC, while the three characteristic peaks at 646.6, 366, and 313 cm^−1^ are credited to lattice vibrations of Mn–O and bending mode vibrations of Mn–O–Mn in the MnO_2_ structure and are aligned with reports in the literature.^[^ ^48^, ^49^, ^50^ ^]^ XPS analysis of the MnO_2_ deposited on C‐cloth was performed; the wide scan survey (Figure S3, Supporting Information) shows peaks from Mn, C, and O elements, and parallel the results from EDX (Figure S4, Supporting Information). Further analysis of the HR‐XPS spectra of Mn 2p, Mn 3s, and O 1s are shown in Figure 2e,f, and Figure S5 (Supporting Information), the corresponding bonding information is listed in Table S2 (Supporting Information). The Mn 2p region shown in Figure 2e comprises spin‐orbit doublet of Mn 2p_1/2_ and Mn 2p_3/2_ observed at 653.99 and 642.39 eV.^[^ ^53^ ^]^ The spin‐energy split of 11.6 eV indicates the presence of MnO_2_.^[^ ^53^, ^54^, ^55^ ^]^ The binding energies of Mn 2p_3/2_ states are obtained at 642.39 and 653.99 eV (Mn^4+^), further confirming the composition of the deposited flower structure contains tetra‐valent Mn in the form of MnO_2_. This is corroborated by the deconvolution of the Mn 3s_1/2_ and 3s_5/2_ peaks at 89.37 and 84.37 eV have a peak split of ≈5 eV, which is representative of MnO_2_.^[^ ^16^ ^]^ The O 1s spectra (Figure S5b, Supporting Information) comprise of an intense peak located at 529.73 eV representing Mn–O–Mn bonds, and a few additional peaks at 531.53, 533.42, and 535.09 eV corresponding to Mn–O–H, C–O and C═O bonds, respectively.^[^ ^9^, ^53^, ^54^, ^55^ ^]^ The C 1s peak situated at 284.79 eV reveals the existence of C─C in the MnO_2_ C‐cloth CC sample, and the absence of any Zn peak in the wide XPS spectra (Figure S3, Supporting Information) further confirms that no Zn is in the cathode structure after a full charge. During charging at 2.2 V, a uniform thin layer of MnO_2_ is coated on the surface of the C‐cloth CC, as observed by FE‐SEM (Figure 2g; Figure S6a–c, Supporting Information). FE‐SEM image with a high‐resolution counterpart, clearly illustrates the uniform deposition of MnO_2_ nano‐flowers on the surface of C‐cloth. Furthermore, we have included a comparison that demonstrates the differences in morphology of the before (high‐resolution image of pristine C‐cloth, Figure S7, Supporting Information) and after the deposited MnO_2_ nanoflowers, providing additional confirmation of the uniform deposition of MnO_2_ nano‐flowers on the C‐cloth CC surface. The high‐resolution SEM images show the agglomerated nano‐flower morphology of the deposited MnO_2_ as illustrated in Figure S6a–c (Supporting Information). Further, HR‐TEM and SAED of the deposited MnO_2_ were carried out and are shown in Figure 2h,i, and Figure S6d (Supporting Information). Figure S6d (Supporting Information) shows the bright field TEM image of deposited MnO_2_ nanoparticles having the interplanar distances of 0.65 and 0.59 nm matching the (211) and (110) planes of *β*‐MnO_2_, respectively (Figure 2h). The concentric circles of the SAED pattern (Figure 2i) confirm the polycrystalline nature of the MnO_2_ nanoparticles, and the obtained planes are consistent with the analysis of the XRD spectra. This investigation confirms the high structural stability of the *β*‐MnO_2_ polymorph deposited on C‐cloth CC under battery cycling conditions, as evidenced in the consistent nature of the discharge plateaus across the first 20 cycles as shown in Figure 1g. The findings align well with the study conducted by You et al., wherein it was demonstrated that *β*‐MnO_2_ exhibits superior thermodynamic stability and a more regular morphological structure during Zn–MnO_2_ battery operation.^[^ ^41^ ^]^

### 2.3 Role of Electrolyte Composition on Cycling Stability

**Figure 3:**
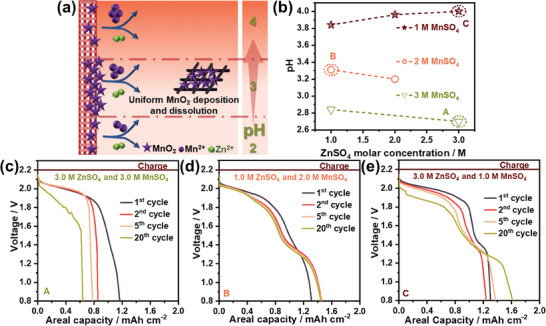
The effect of electrolyte pH and relative Mn^2+^ and Zn^2+^ ion concentrations on the electrochemical performance of Zn–MnO_2_ batteries. a) Schematic representation of the effect of changing pH on the deposition and dissolution of MnO_2_ on a C‐cloth CC. b) The change in pH of electrolyte with variance in the molar ratio of ZnSO_4_ and MnSO_4_ from 1 to 3 m. The changes in the discharge curves at a discharge current rate of 6 mA cm^−2^, cell voltage, and areal capacities with changing electrolyte pH and concentration. The nature of discharge curves at c) 3 m ZnSO_4_ and 3 m MnSO_4_ with a pH of 2.70, d) 1 m ZnSO_4_ and 2 m MnSO_4_ with a pH of 3.31, and e) 3 m ZnSO_4_ and 1 m MnSO_4_ and with a pH of 4.00. The lower the pH of the electrolyte the more elongated the discharge curves.

**Figure** **3**a‐b shows the effect of the concentration of ZnSO_4_ and MnSO_4_ on the pH of electrolytes, and shows that changing the concentration of Zn^2+^ and Mn^2+^ between 1 to 3 m results in a change in pH from 4.00 to 2.70 (classified into low, medium, and high pH). Keeping the concentration of Mn^2+^ at 1 m and varying the Zn^2+^ concentration from 1 to 3 m, the pH increased from 3.84 to 4.00, whereas, inversely, keeping the Zn^2+^ concentration at 1 m and changing the concentration of Mn^2+^ from 1 to 3 m the observed pH values decreased from 3.31 to 2.84 (Figure 3b). Overall, we see that the pH of the electrolyte is a stronger function of the concentration of Mn^2+^ than it is of Zn^2+^. In the same figure (Figure 3b), we have noted some exemplar electrolyte compositions that are described to demonstrate the general trends in battery performance (Figure 3c–e; Figure S8a–c, Supporting Information). It was observed that electrolytes with low pH, but high concentration (Figure 3c) help to stabilize the high voltage discharge plateaus,^[^ ^9^ ^]^ but also lead to a decrease in areal capacity in the subsequent cycles from the saturation and precipitation of electrolytes at high concentrations, as observed in the electrolyte image in Figure S9 (Supporting Information). In Figure 3d,e we show the discharge curve for lower overall concentrations of the electrolyte both electrolytes demonstrate elongated voltage plateaus. Figure 3d and Figure S8b (Supporting Information) shows that with lower Zn^2+^ proportions, a more cycle‐stable discharge curve with a less prominent low‐voltage discharge plateau is achieved. Electrolyte B, characterized by a high concentration of MnSO_4_ and elevated pH, contains both abundant H^+^ species and a substantial Mn^2+^ concentration.^[^ ^14^ ^]^ In the initial step, hydrogen ions (H^+^) adsorb onto the O site of the deposited MnO_2_, forming an OH^‐^ group. The subsequent step involves H^+^ adsorption on a neighboring Mn site. Subsequently, water is generated through the combination of the adsorbed H^+^ with OH^‐^ which establishes a favorable equilibrium between the dissolution of Mn^2+^ from the C‐cloth to the electrolyte and the concurrent re‐oxidation of Mn^2+^ in the electrolyte, thereby facilitating continuous dissolution/oxidation of Mn^2+^/MnO_2_ reactions. This contributes to enhancing cyclic stability and mitigating capacity fading issues by propagating higher utilization of MnO_2_ active material.^[^ ^61^ ^]^ A similar observation of a longer high voltage discharge plateau and the disappearance of the lower voltage plateau is made at pH 2.7 (where Zn^2+^ and Mn^2+^ are at equivalent molar concentrations of 3:3) that further confirms the competitive reaction of protons, Zn^2+^, and Mn^2+^ with MnO_2_ at discharge. However, at high concentrations, the saturation of the electrolyte (Figure S9c, Supporting Information) results in a decrease in the areal capacities over the first 20 cycles. This observation suggests that a large number of protons drive the electrochemical reaction toward MnO_2_ dissolution, which results in a low overpotential.^[^ ^14^, ^62^, ^63^, ^64^, ^65^ ^]^ In principle, Mn^2+^ ions could suppress MnO_2_ dissolution into the electrolyte, thus improving the stability of the cathode and utilization of active material.^[^ ^29^, ^30^, ^31^, ^32^ ^]^ However, once the Mn^2+^ has been consumed from the electrolyte, the capacity fades and thus, high concentrations of Mn^2+^ in the electrolyte are not effective for long‐term cycling.^[^ ^64^, ^65^ ^]^ Critical to MnO_2_ cathodes is the modulation of Mn^2+^ ion concentration. Arising from active material dissolution and preinclusion in the electrolyte, Mn^2+^ generation induces pH alterations, impacting MnO_2_ charge/discharge electrochemistry resulting in the reversible deposition and dissolution reaction and diminishing the parasitic reactions. Hence, an electrolyte relatively modest in Mn^2+^ with a controlled pH is required to enhance the electrochemical reaction over long‐term cycling, as exemplified by electrolyte B.

### 2.4 Battery Prototype Evaluation

**Figure 4:**
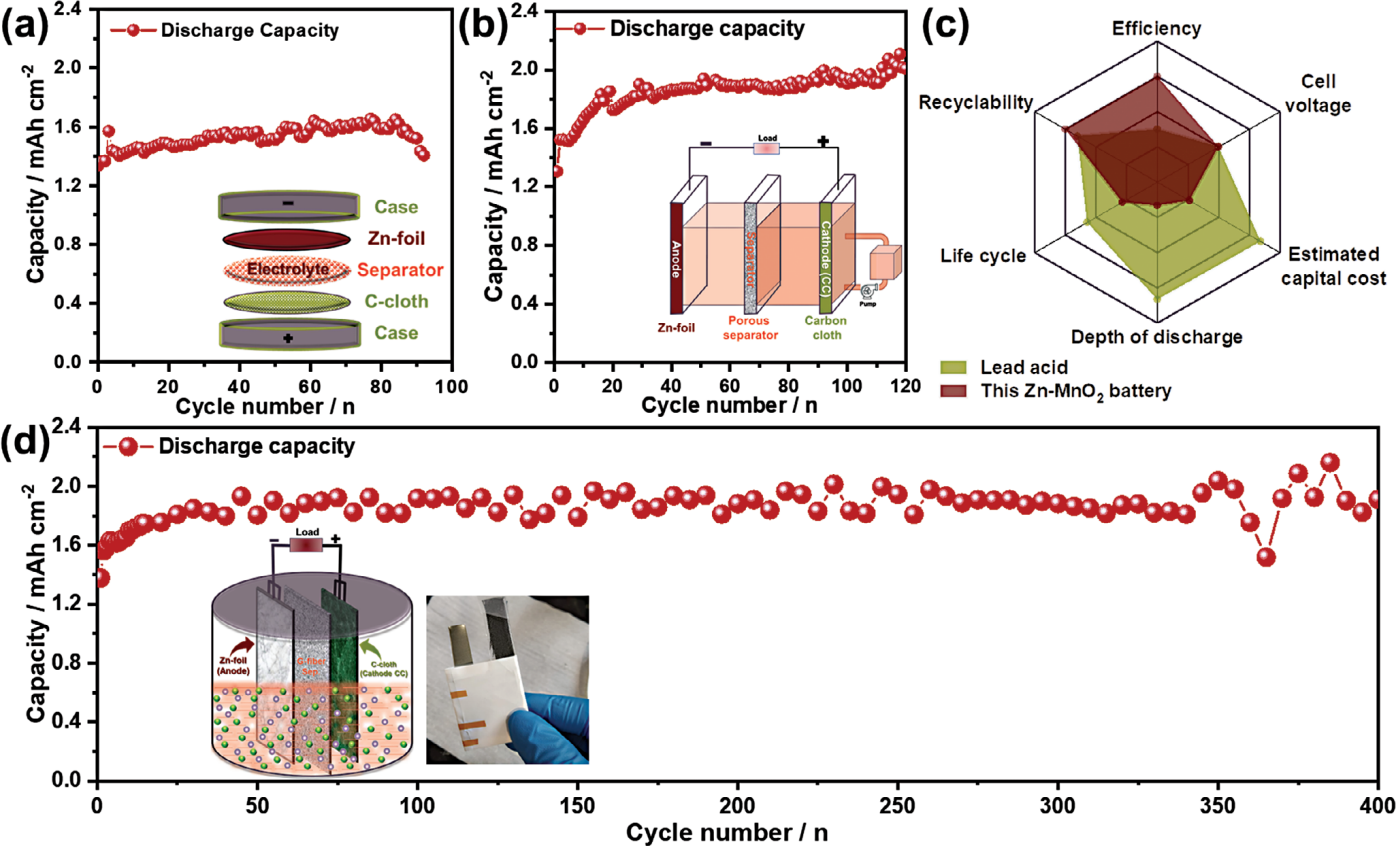
Zn–MnO_2_ battery prototype architectures. The electrochemical performance of Zn–MnO_2_ battery in a) a 2032 type coin cell configuration, b) a semi‐flow cell configuration, and d) a novel flooded stack‐cell type architecture with a schematic of the cell configuration in the insets of the respective figures. c) Comparative analysis of the sustainability of Zn–MnO_2_ against lead‐acid batteries in terms of efficiency, cell voltage, estimated capital cost, depth of discharge, life cycle, and recyclability. Literature data is available in Table S3 (Supporting Information). d) The long‐term cycle performance of Zn–MnO_2_ battery over 400 cycles at a discharge current rate of 10 mA cm^−2^.

Researchers have explored numerous cell designs to enhance understanding of cell behavior in scalable architectures, focusing on the impact of optimized electrolyte chemistries and CC on the specific energy and power density in aqueous Zn–MnO_2_ batteries.^[^ ^44^ ^]^ The investigation involves varying the area and shape of electrodes and separators to identify optimal configurations. Herein, distinct cell setups, including coin cells, flow cells, and flooded electrode‐stack architectures, have been employed to assess their effects on capacity and rate. These variations influence electrolyte access, leading to substantial differences in the utilization of MnO_2_ active material. Initially, battery performance was examined using a coin cell design (**Figure** **4**a). However, after a few initial cycles, the cell expanded due to the generation of high internal stress from gas formation during the charge/discharge process.

Thus, more optimal designs were needed for the venting of these gases during cycling, and therefore a flow cell design was investigated, as shown in Figure 4b. In this setup, Zn foil only requires access to the electrolyte on the side facing the C‐cloth CC. The semi‐flow design ensures the circulation of electrolytes within the CC, requiring only a single pump for full‐cell operation. The semi‐flow cell design holds the advantage of minimizing power requirements for pumping electrolytes in the Zn–MnO_2_ battery, contributing to cost‐efficient operation.^[^ ^66^ ^]^ While the semi‐flow cell demonstrated satisfactory performance for ≈100 cycles, issues emerged over time. The formation of Zn dendrites and corrosion of the Zn anode became apparent, leading to a gradual capacity loss after 120 cycles and ultimately resulting in cell failure.

The more advanced cell design adopted was the stack cell design featuring a flooded cathode, anode, and separator. The inset of Figure 4d shows the schematic representation of the flooded stack cell Zn–MnO_2_ battery design. This design stands out for its simplicity, scalability, and lack of requirement for a customized setup, aligning with the prevalent technology seen in the market for LABs. Flooded architectures, similar to those employed in LABs, offer distinct advantages. Flooded batteries, characterized by an open architecture, allow for overcharging with a reduced risk of damage compared to sealed batteries. They exhibit fewer issues with stresses induced by battery operation.^[^ ^67^ ^]^ In this configuration, the open architecture facilitates the release of all gas generated during cycling. However, it is crucial for the interfaces between stacked materials to be robust and exhibit chemical and mechanical compatibility. This consideration is paramount for ensuring the overall effectiveness and longevity of this cell design.^[^ ^67^ ^]^

The flooded stack cell aqueous Zn–MnO_2_ battery showed excellent cycling stability over 400 cycles with a capacity retention of ≈100% at a discharge current rate of 10 mA cm^−2^, as shown in Figure 4d with an interval of 5 cycles per data point. The Galvanostatic discharge plateaus of the 1st to 30th cycles are shown in Figure S10 (Supporting Information). The elongated plateau occurs at ≈2 V and the discharge capacity reaches ≈2 mAh cm^−2^ with a capacity retention of ≈100%, which confirms that the voltage window of the battery has increased, a benefit of operating these battery chemistries in optimized low pH electrolytes.^[^ ^14^ ^]^ The procedure adopted by Li et al. has been employed to calculate the CE.^[^ ^9^ ^]^ The CE for the long‐term cycle performance of 2032 type coin cell configuration, semi‐flow cell configuration, and flooded stack‐cell type architecture is provided in the Supplementary information as Figure S11a–c (Supporting Information). Additionally, the cycling stability tests of the flooded stack‐cell type architecture demonstrate a CE ranging from 80% to ≈100% over 400 cycles. This variation further validates the effective depositing and dissolving of Mn^2+^ ions during battery operation. Furthermore, Figure S12 (Supporting Information) depicts the rate performance of the flooded stack‐cell at 2, 6, 10, 15, and 20 mA cm^−2^, showing consistent discharge capacities, especially at 10 mA cm^−2^ (5C, a rate of nC corresponds to a full discharge in 1/nh,).^[^ ^9^ ^]^ This underscores the ability of the stack‐cell Zn–MnO_2_ battery to operate effectively, even under high‐rate conditions, demonstrating its suitability for various discharge rates. Zn–MnO_2_ batteries can be a potential alternative for LABs. A comparative analysis of the sustainability of Zn–MnO_2_ and LABs is presented in Table S3 (Supporting Information) revealing that this Zn–MnO_2_ batteries have a specific energy density (673 Wh kg^−1^, considering the weight of the deposited MnO_2_ active material, which is consistent with reported literature)^[^ ^14^ ^]^ approximately tenfold higher than LABs, higher recyclability, a cell voltage (≈2 V) comparable to LABs, and an estimated cost (≈US$10/ kg, detailed calculation given in the Supporting Information) much lower than LABs. The electrochemical data presented in Table S4 (Supporting Information) reveals enhancements in efficiency, overall cell voltage, cycle number, and capacity compared to the literature. These findings underscore the relevance and suitability of this work in the domain of Zn–MnO_2_ batteries. This comparison further affirms the advantages of adopting Zn–MnO_2_ batteries for stationary storage applications, due to their low capital cost, use of sustainable materials, and environmentally friendly chemistry. This high voltage Zn–MnO_2_ battery utilizing an optimized electrolyte could become an inexpensive alternative from the perspectives of manufacturing and raw material abundance, an ecologically sound option for storing energy from renewable sources, and a strong candidate as a high‐energy density solution for storing renewable energy and for integration into the grid.

### 2.5 Post‐Mortem Analysis

To further delineate the failure mechanism that is occurring within the battery, a post‐mortem analysis was conducted following the charge/discharge state at a voltage range of 0.8–2.2 V. As observed in the FE‐SEM image, shown in Figure S13a (Supporting Information), there is a uniform coating of MnO_2_ on the C‐cloth and a high‐resolution view shows the flower‐type morphology of deposited MnO_2_. EDS mapping (Figure S13b, Supporting Information) confirms the uniform distribution of Mn and O elements on the C‐cloth surface. At a discharge voltage of ≈1.6 V (Figure S13c‐d, Supporting Information), the slow dissolution of deposited MnO_2_ has initiated (the corresponding EDS mapping is shown in Figure S13d, Supporting Information); at 1.2 V, the majority of deposited MnO_2_ has dissolved into the electrolyte system as Mn^2+^, leaving discrete patches of solid MnO_2_ (Figure S13e, Supporting Information). At full discharge, 0.8 V, the remaining patches of MnO_2_ have dissolved, leaving a negligible fraction of MnO_2_, as seen in Figure S13f (Supporting Information).^[^ ^9^, ^43^ ^]^ The other critical observation lies in the reaction at the Zn anode. Figures S14 and S15 (Supporting Information) show a comparison of SEM images of Zn electrodes before and after cycling, prior to cycling the Zn plate exhibits a smooth, dense surface, which would be very important for the long‐term cycling stability of Zn–MnO_2_ batteries. After 400 cycles, no noticeable change in the morphology of the carbon cloth CC is observed, but some fraction of deposited MnO_2_ remains on the C‐cloth surface (Figure S17, Supporting Information. Post‐analysis of the Zn foil anode shows a flat surface of the pristine Zn foil (Figure S14, Supporting Information) which changed into a mossy growth of nanoflakes following Zn dendrite growth after 100 cycles, as shown in Figure S16a–d (Supporting Information). The EDS mapping of the post‐cycled anode, as shown in Figure S16d (Supporting Information), confirms the presence of only Zn and O.^[^ ^9^, ^43^ ^]^ The Zn foil corrodes fully following more than 400 cycles as seen in Figure S17 (Supporting Information). Another potential cause for dendrite growth is the often‐overlooked influence of the surface structure of commercially used zinc foils in Zn–MnO_2_ research.^[^ ^16^, ^68^ ^]^ The surface irregularities introduced during manufacturing, including rough textures, microcracks, scratches, fold lines, or unpolished edges, serve as defect sites conducive to local dendrite growth.^[^ ^16^ ^]^ Exploring simple, scalable, and effective methods, such as polishing and coating, to rationally modify the surface of pristine zinc can enhance the overall performance of the cell. Recent studies illustrate that precise topographical control, achieved through a straightforward and scalable acid treatment applied to zinc foils, allows for the deposition of zinc at remarkably low overpotentials, ensuring ultra‐stable performance.^[^ ^68^ ^]^ Implementing these approaches has proven helpful in facilitating uniform zinc deposition through reducing the uncontrolled accumulation of side‐reaction products on the untreated zinc anode. Consequently, they may contribute to maintaining excellent stability, shedding light on further studies, and paving the way for real‐life applications.^[^ ^68^ ^]^ While this studies demonstrate that the utilization of a low pH electrolyte for efficient deposition of MnO_2_ and subsequent cycling is a step in the right direction, further protection of the anode is necessary to take full advantage of the high voltage window and enhance the long‐term cycle life of the aqueous Zn–MnO_2_ battery.

## 3 Conclusion

In summary, a high‐voltage prototype of an aqueous Zn–MnO_2_ battery was successfully developed, utilizing metallic Zn foil as the anode and electrochemically deposited MnO_2_ as the cathode within a flooded stack cell configuration. The charging process was confirmed to result in the deposition of Mn^2+^ on the cathode CC in the form of *β*‐MnO_2_, with reversible dissolution into the electrolyte during discharge. A comparative analysis of various carbon sources indicated that an optimized aqueous Zn–MnO_2_ battery employing C‐cloth as the CC can achieve a cell voltage of ≈2.00 V. An electrolyte lean in Mn^2+^, with controlled pH, enables uniform MnO_2_ deposition, avoids generating electrochemically inactive species and facilitates operation over a larger voltage window and stable performance over long‐term cycling. This configuration exhibited superior capacity retention of ≈100% even at a high current rate of 10 mA cm^−2^. The scalability of absolute energy output was evident through the remarkable stability of capacity observed over 400 cycles, showcasing significant potential for large‐scale energy storage applications. The study suggests that further optimization could be achieved by designing specialized current collectors with defect‐rich high surface areas and high ionic conductivities to facilitate the rapid deposition and dissolution of active materials. Overall, this work establishes a robust foundation for the development of the next‐generation, low‐cost, and safe energy storage system tailored for grid‐scale applications.

## 4 Experimental Section

### Materials and Methods: Zn–MnO_2_ Battery Assembly

The Zn–MnO_2_ electrochemical cells were fabricated using commercially available zinc foil (300 mm × 300 mm, thickness 0.1 mm, Sigma–Aldrich) as the anode. The cathode was formed in situ through direct electrodeposition on carbon‐based CCs such as C‐cloth, CNTs, and G‐felt, (1071 HCB, CT GF020, Fuel Cell Store, Australia) in a technique developed by Li et al.^[^ ^9^ ^]^ Whatman glass microfiber (Sigma–Aldrich) was used as the separator. The cathode CCs were treated in plasma for 5–30 min before the cell fabrication to increase hydrophilicity and introduce more active sites for electrolyte access. Subsequent structural and chemical analysis was conducted on the plasma‐treated CCs.

### pH‐Engineered Electrolyte

The electrolyte had been synthesized from zinc sulphate monohydrate (ZnSO_4_·H_2_O, ≥99.0%, Sigma–Aldrich) and manganese sulphate monohydrate (MnSO_4_·H_2_O, ≥99.0%, Sigma–Aldrich) in concentrations ranging from 1 to 3 m MnSO_4_ and 1 to 3 m ZnSO_4_ in ratios of 1:3 to 3:1 Mn^2+^ to Zn^2+^. The pHs of these electrolyte systems were measured to be between pH 2–4 using Hanna Instruments pH meter.

### Cell Assembly

Aqueous Zn–MnO_2_ cells were assembled in coin‐cell (CR2032), flow cell, and simple stack‐type cell architectures to illustrate the realistic and scalable performance of these batteries.

### Flow Cell Assembly

The single zero‐gap flow cell hardware includes the Zn anode and C‐cloth CC separated by the glass fiber separator where electrolyte was driven across the CC by a peristaltic pump (BT300‐2J Longer Precision Pump Co., Ltd.) at a flow rate of 10 mL min^−1^ at room temperature. The 10 mL electrolyte reservoirs were connected to Bio Logic Potentiostat for electrochemical testing.

### Simple Stack‐Type Cell

The Zn foil, C‐cloth CC, and separator were cut into 3 cm × 3 cm pieces with a protrusion of 1 cm × 1 cm for electrical connection to the Potentiostat. These pieces were stacked together and then bound by celgard to ensure good contact throughout the experiment (inset of Figure 4d). After stacking, the stack was transferred into a glass cell containing the electrolyte. Where all cell components (cathode CC, anode, and separator) were operated in a flooded configuration (Figure S18, Supporting Information).

### Electrochemical Tests

Chronoamperometric charge and Galvanometric discharge, measurements of aqueous Zn–MnO_2_ batteries were recorded using Biologic VSP3e Potentiostat (BioLogic Science Instruments, France) and BT‐Lab battery testing equipment (BioLogic Science Instruments, France) at room temperature.

### Materials Characterization

Morphology studies of the MnO_2_ active material formed by the electrodeposition on carbon cloth have been investigated by SEM, and TEM, and structural characterization was carried out by XRD, Raman, and XPS techniques.

Scanning electron microscopy (FEI Nova Nano SEM 450 FEG and Thermo Scientific Verios 5 UC FEGSEM) was used to investigate the morphology of carbon sources, deposited MnO_2_ nanoparticles, and at different depths of discharge. For EDS spectra, the secondary electron images were collected at 15 kV.

Transmission electron microscopy (FEI Tecnai G2 T20 TEM) was used to analyze the structure of deposited MnO_2_. The deposited MnO_2_ sample was dispersed in ethanol and mounted on a copper grid. The TEM samples were investigated at an operating voltage of 120 keV.

X‐ray diffraction (Bruker D8 Advance Eco Diffractometer XRD) was conducted using Cu K*α* radiation generated at 40 kV and 10 mA at a scan rate of 0.2 min^−1^ with a step size of 0.01 to investigate the structural changes in the three types of carbon sources and confirm the deposition of MnO_2_ on the C‐cloth.

X‐ray Photoelectron Spectroscopy (Thermo Fisher Scientific Nexsa Surface Analysis System) was conducted to investigate the C 1s and O 1s peaks of the three carbon sources and after the deposit of MnO_2_ on C‐cloth using a monochromatic Al K*α* source.

Raman spectra (Renishaw Confocal micro‐Raman Spectrometer) were recorded using a 514 nm wavelength of argon laser working at 10% power between 100 cm^−1^ to 3500 cm^−1^ wavenumbers.

## Conflict of Interest

The authors declare no conflict of interest.

## Author Contributions

The manuscript was written through the contributions of all authors. All authors have approved the final version of the manuscript.

## Supporting information

Supporting Information

## Data Availability

The data that support the findings of this study are available from the corresponding author upon reasonable request.
