# Supplementary material for: Acidity‐Aided Surface Modification Strategy to Enhance In Situ MnO2 Deposition for High Performance Zn‐MnO2 Battery Prototypes
Source: Small. 2024 Mar 28;21(28):2311933. doi: 10.1002/smll.202311933 (PMC12272039; doi:10.1002/smll.202311933)
Supplement: Supplementary file 1 — Supporting Information [file SMLL-21-2311933-s001.pdf]

NANO · MICRO

small

Supporting Information

for *Small*, DOI 10.1002/smll.202311933

Acidity-Aided Surface Modification Strategy to Enhance In Situ MnO_2 Deposition for High Performance Zn-MnO_2 Battery Prototypes

Manas Ranjan Panda, Sally El Meragawi, Meysam Sharifzadeh Mirshekarloo, Wanqing Chen, Mahdokht Shaibani and Mainak Majumder**

Supporting Information

Acidity-aided Surface Modification Strategy to Enhance In-situ MnO_2 Deposition for High Performance Zn-MnO_2 Battery Prototypes

Manas Ranjan Panda[1,2], *Sally El Meragawi*[1,2], *Meysam Sharifzadeh Mirshekarloo*[1], *Wanqing Chen*[1], *Mahdokht Shaibani*[2,3*], *Mainak Majumder*[1,2*]

[1]Nanoscale Science and Engineering Laboratory (NSEL), Department of Mechanical and Aerospace Engineering, Monash University, Clayton, VIC, 3800, Australia

[2]ARC Research Hub for Advanced Manufacturing with 2D materials (AM2D), Monash University, Clayton, VIC, 3800, Australia

[3]Department of Chemical and Environmental Engineering, RMIT University, Melbourne, VIC, 3001, Australia

[*]Corresponding authors: Mainak Majumder (mainak.majumder@monash.edu) and Mahdokht Shaibani (m.e.shaibani@monash.edu)

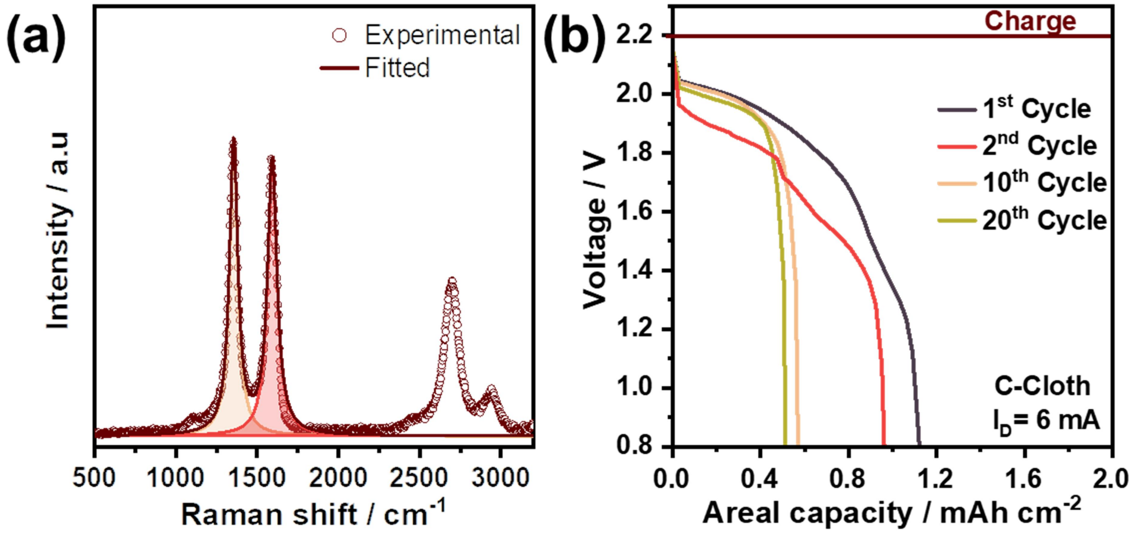

Figure S1. (a) Raman spectra and (b) the variation in the discharge curves, cell voltage, and areal capacities of pristine C-cloth current collector (CC).

Methanol washing and plasma treatment were applied to three commercially available carbon materials. Raman characterization was used to validate defect creation at various plasma treatment durations, between 5 to 30 minutes, for the optimized CC (C-cloth) and compared it to the untreated C-cloth CC. Figure S1 and Figure 1 illustrates the correlation between the structures of pristine and plasma-treated C-cloth CCs and their respective electrochemical performances. The I_D/I_G intensity ratio for the plasma-treated C-cloth is 1.18 and is higher than that of pristine C-cloth CCs (I_D/I_G intensity ratio of 1.09), indicating a higher occurrence of defects following treatment. The increased structural disorder and number of defects enhances the adsorption of Mn^{2+} ions into the host structure.[1-11] The XPS spectra of C-cloth CC sample showed high C–O content, further confirming that the percentage of oxygen-containing functional groups may be the source of the high defect count observed in Raman and thus represent a higher number of active sites for the robust deposition of Mn^{2+} on the surface of C-cloth and G-felt CCs.[6-9, 11] The plasma-treated C-cloth demonstrates superior electrochemical performance in terms of both cycling stability and capacity compared to the

pristine C-cloth CC. The corresponding electrochemical results are shown in supplementary Figure S1 and Figure 1.

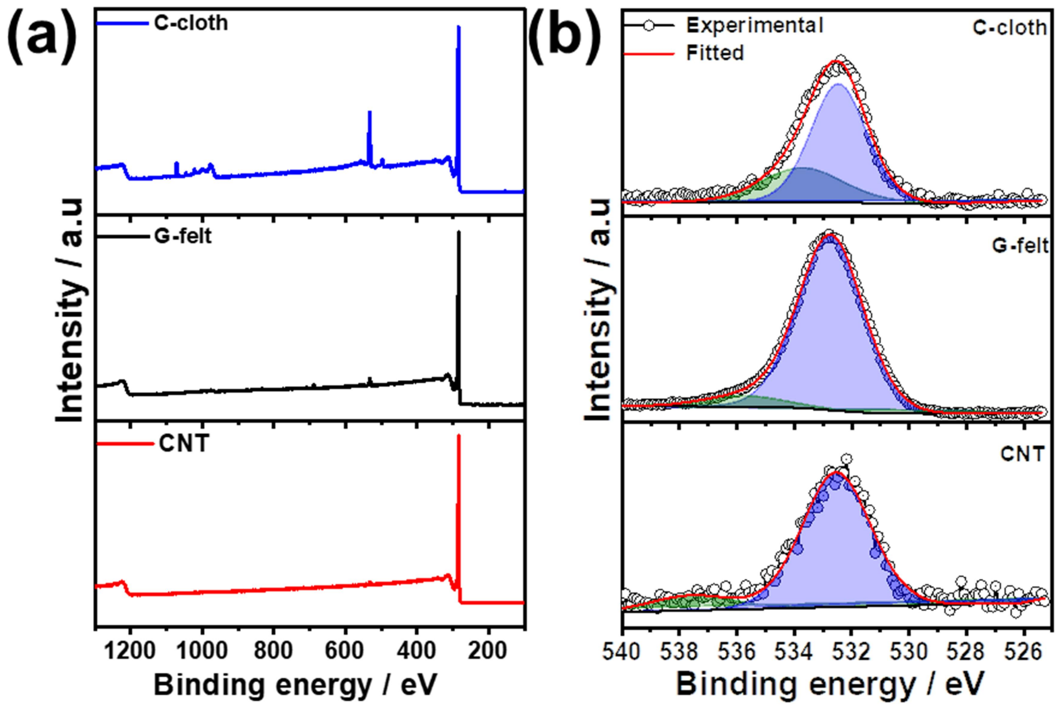

Figure S2. (a) Wide scan survey XPS spectra showing the presence of C and O elements and O1s (b) spectra of C-cloth, G-felt, and CNT CCs samples.

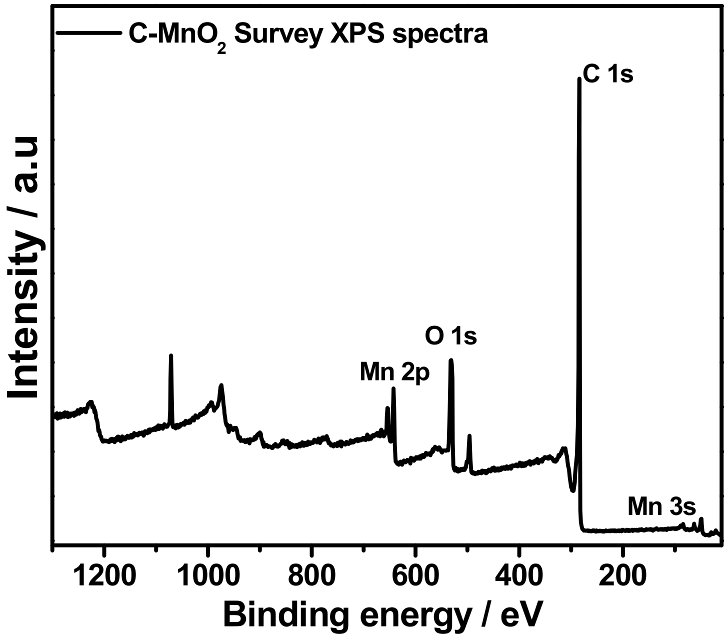

Figure S3. Wide scan survey XPS spectrum of MnO_2 deposited C-cloth CC sample showing the presence of C, O and Mn elements.

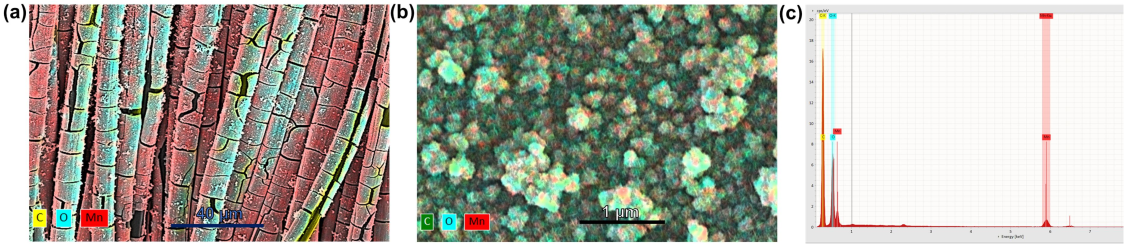

Figure S4. SEM image with EDS mapping of MnO_2 deposited C-cloth.

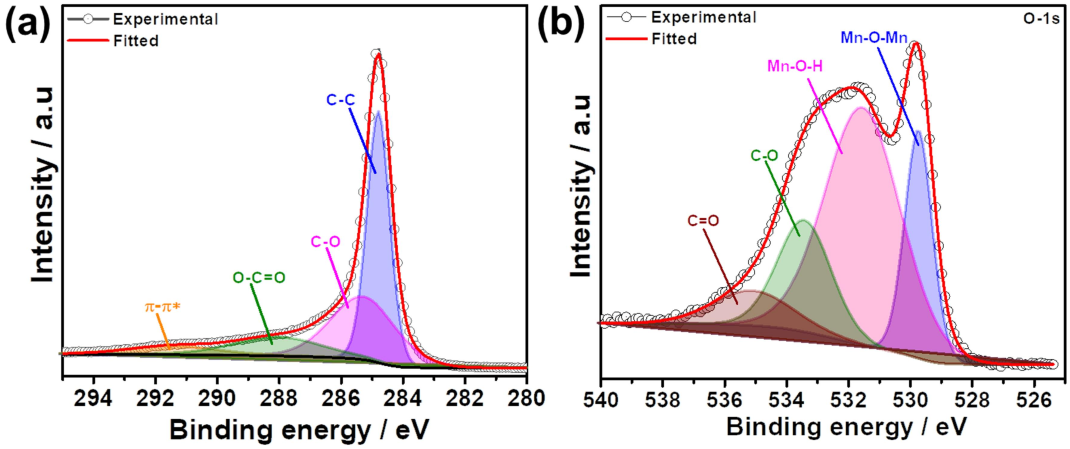

Figure S5. HR-XPS spectra of (a) C 1s and (b) O 1s of MnO_2 deposited on C-cloth after full charge.

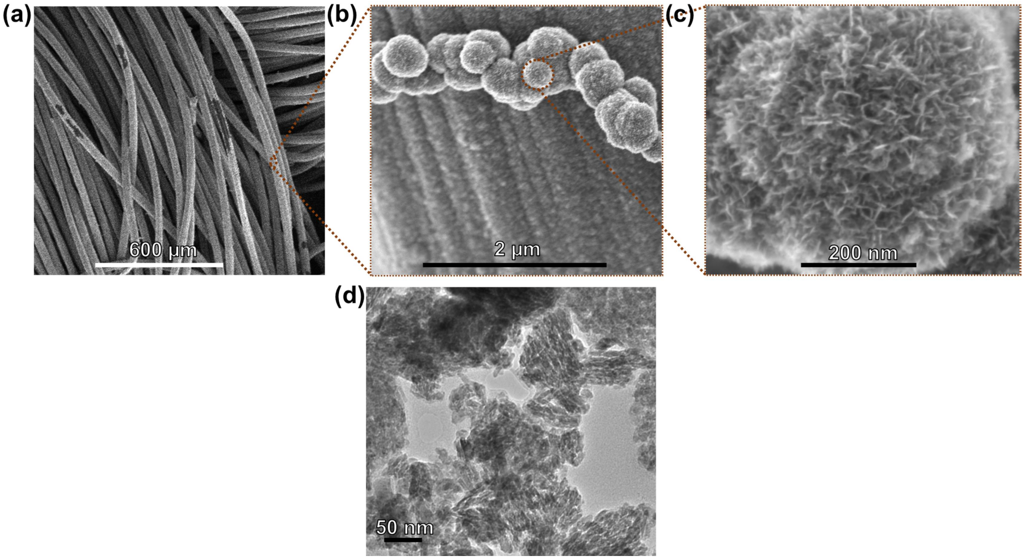

Figure S6. (a-c) Field emission scanning electron microscopy (FE-SEM) image showing the uniform deposition of MnO_2 on the surface of the C-cloth and the corresponding high

magnification images illustrating the flower-like morphology of the deposited MnO_2. (d-f) The HR-TEM image.

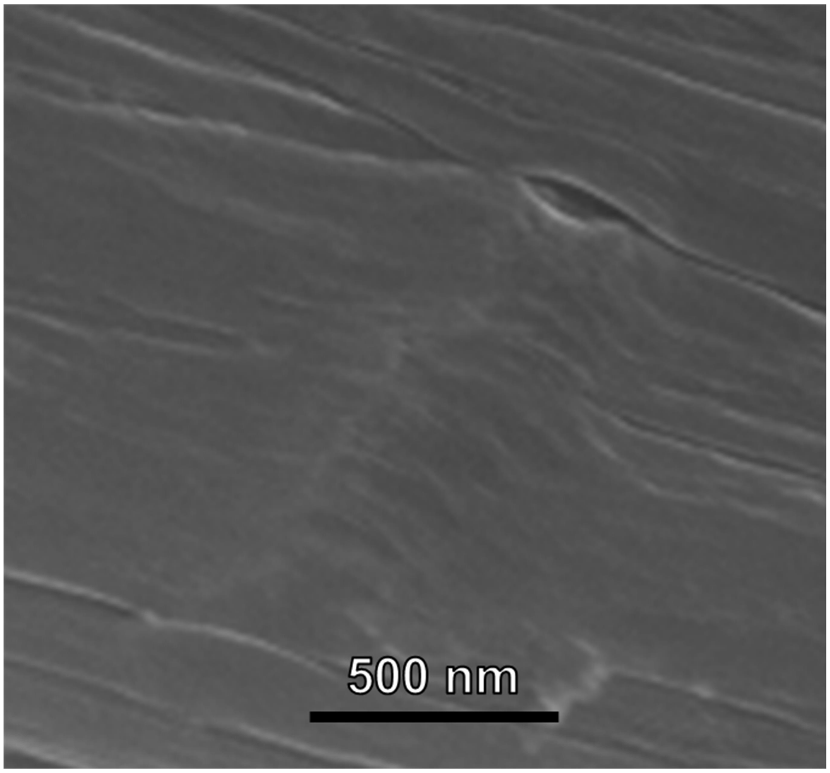

Figure S7. High-resolution FE-SEM of the morphology of pristine C-cloth CC.

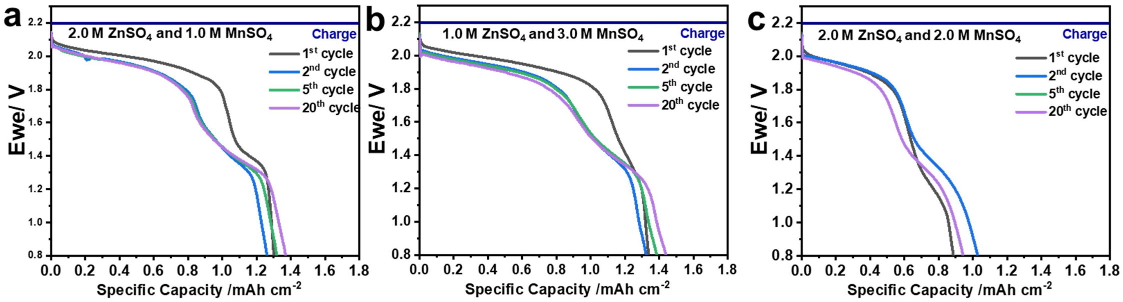

Figure S8. (a) Nature of discharge curves at 1 M $MnSo_4$ and 2 M $ZnSO_4$ with a pH of 3.96. (b) 3 M $MnSO_4$ and 1 M $ZnSO_4$ with a pH of 2.84. (c) 2 M $MnSO_4$ and 2 M $ZnSO_4$ with a pH of 3.20.

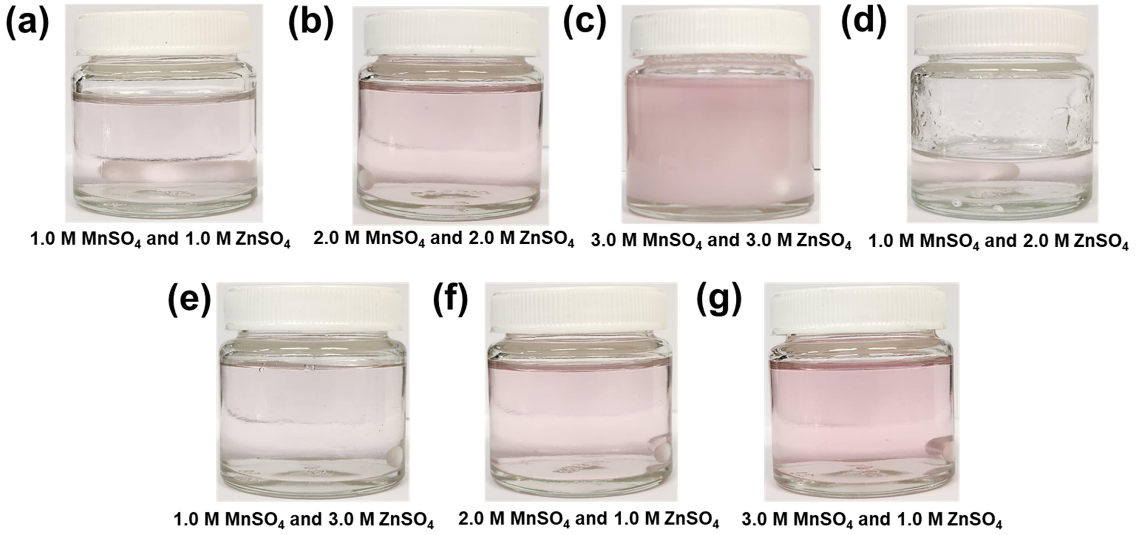

Figure S9. Photographs of electrolytes with different concentrations of Zn^{2+} and Mn^{2+} used in $ZnMnO_2$ battery.

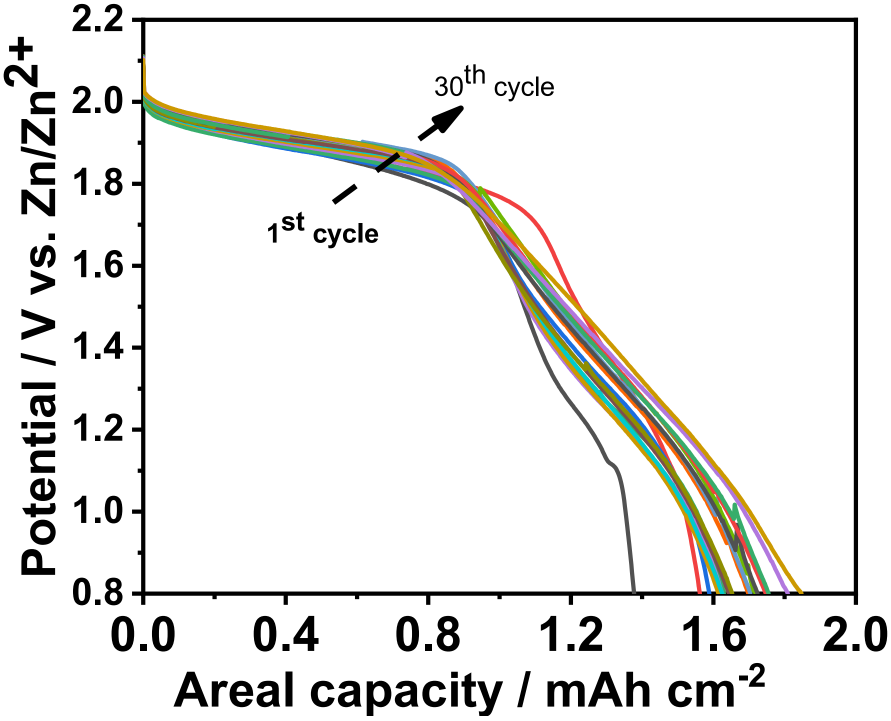

Figure S10. The Galvanostatic discharge plateau of the flooded stack-cell architecture.

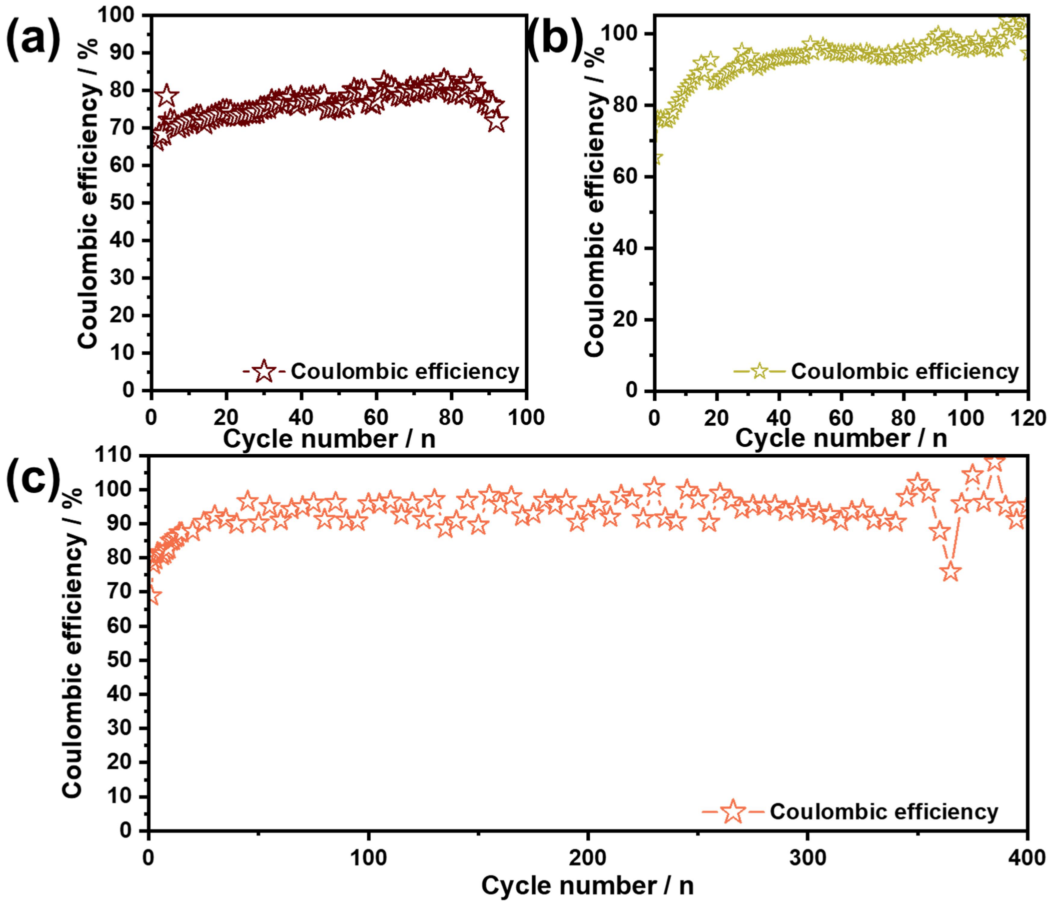

Figure S11. Coulombic efficiency during long-term cycle performance for (a) a 2032 type coin cell configuration, (b) in the semi flow cell configuration, and (c) the flooded stack-cell type architecture over 400 cycles at a discharge current rate of 10 mA cm^{-2}.

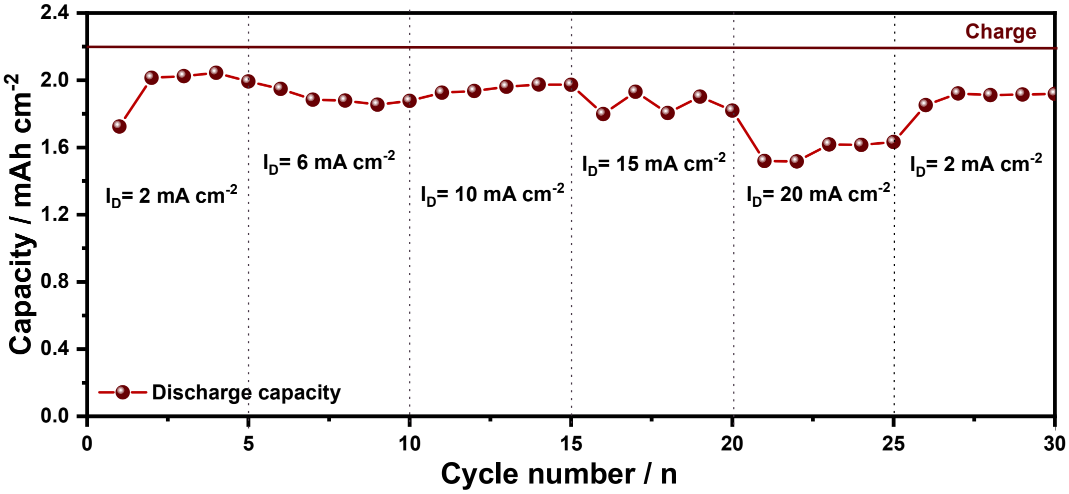

Figure S12. Rate capability at various rates from 2 to 20 mA cm^{-2} of flooded stack-cell architecture Zn-MnO_2 battery.

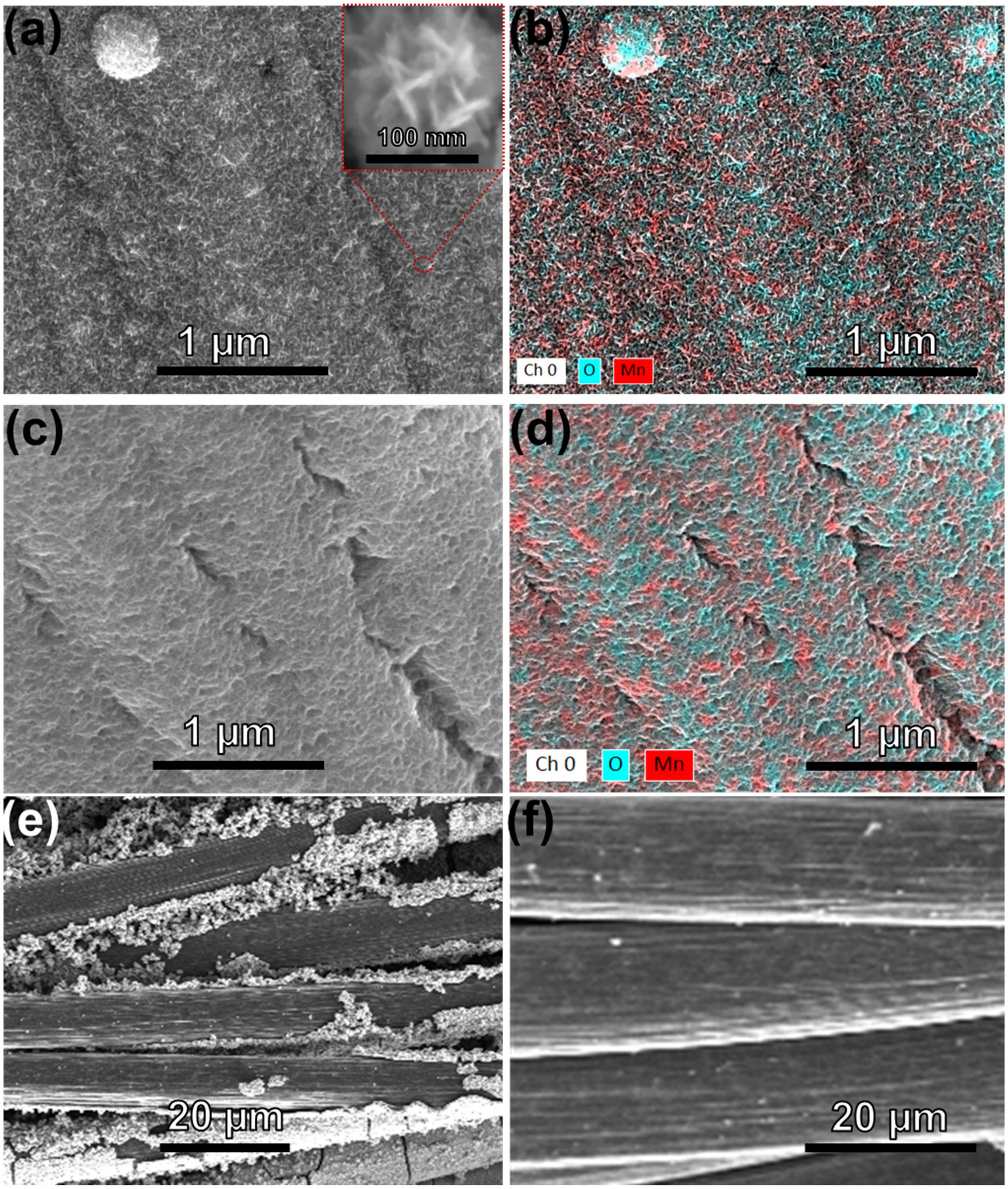

Figure S13. FESEM and EDS images showing the evolution of MnO_2 deposition and dissolution reactions, which occur at full charge/discharge. (a-b) FE-SEM and EDS mapping of a thin layer of MnO_2 deposited at a charge voltage of 2.2 V. (c-d) FE-SEM and EDS mapping of the dissolved MnO_2 at a discharge voltage of 1.6 V. (e-f) FE-SEM images showing patches of MnO_2 remain at 1.2 V and after full discharge states.

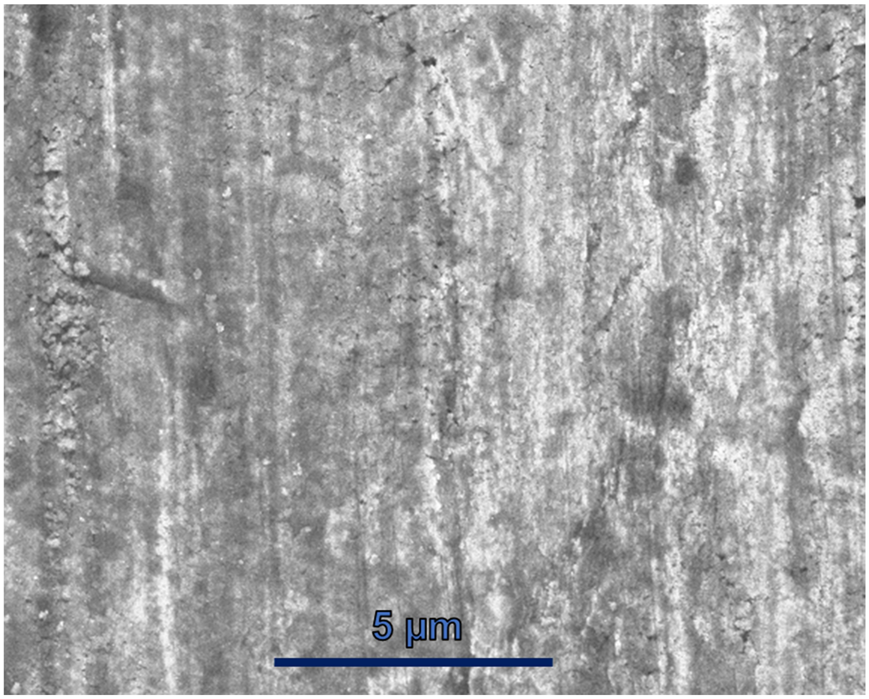

Figure S14. SEM image of bare Zn-foil before electrochemical cycling.

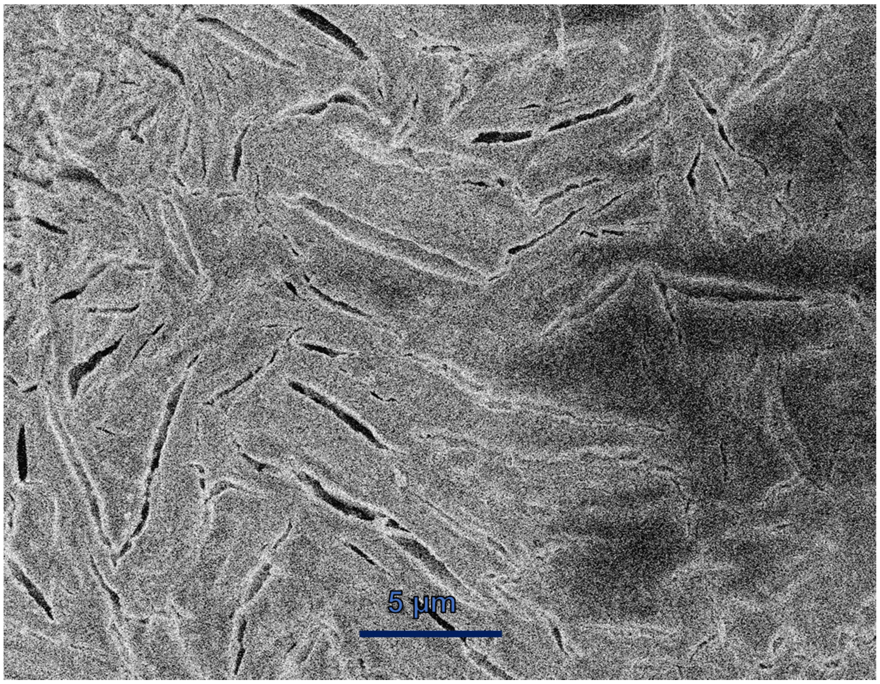

Figure S15. SEM image of Zn-foil after 100 cycles.

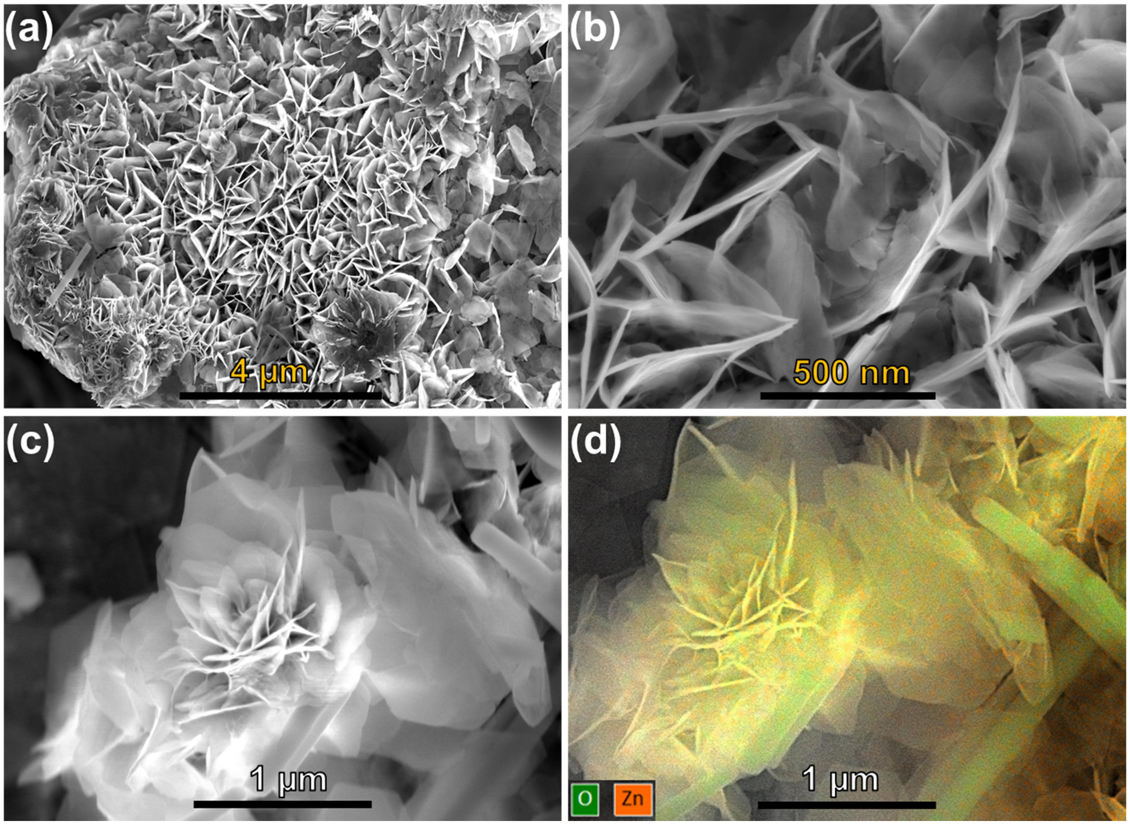

Figure S16. (a-c) FE-SEM images showing morphological change of post-cycled Zn foil anode and the corresponding (d) EDS mapping.

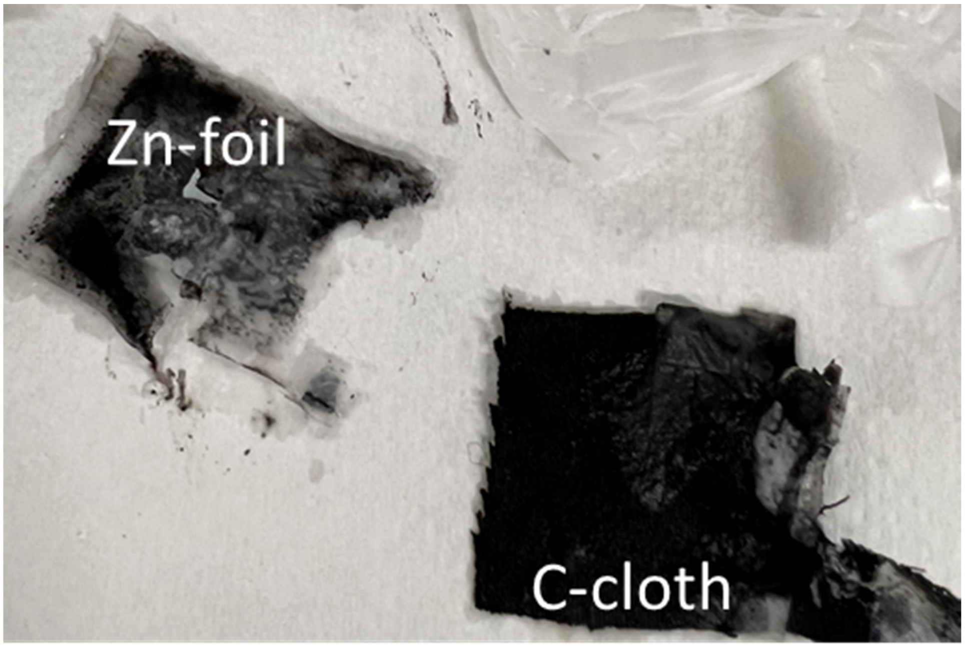

Figure S17. Morphological change of post-cycled Zn foil anode and the C-cloth CC after 400 cycles.

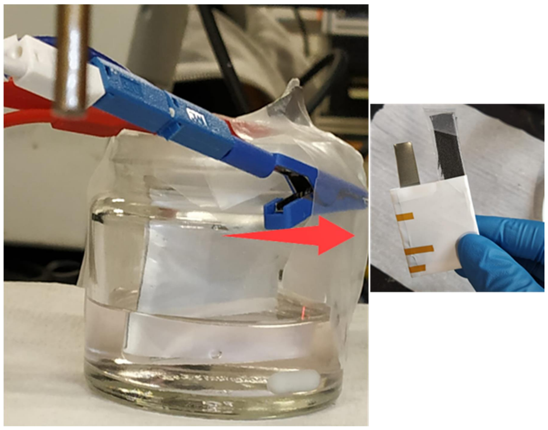

Figure S18. The novel flooded stack-cell type Zn-MnO_2 prototype.

Table S1. Bonding information of XPS spectra of C-cloth, G-felt, and CNT samples as shown in the XPS spectra in Figure 1f and Figure S2b.

**C 1s bonding information**	**C-cloth**	**G-felt**	**CNT**
Sp2(C=C)	284.07	284.03	284.08
C-C	284.76	284.77	284.88
C-O	285.94 (13%)	285.96 (9%)	286.08 (6%)
O-C=O	287.48	287.16	286.98
Π-Π*	290.97	290.23	291.36
**O 1s bonding information**	**C-cloth**	**G-felt**	**CNT**
O-C	537.57		
C=O	532.54	532.73	532.45
O-C=O		535.78	533.71

Table S2. Bonding information of C 1s, O 1s, Mn1s and Mn3p CNT XPS spectra of as grown MnO_2 materials on C-cloth as shown in the XPS spectra in Figure 2e, f and Figure S5.

**C 1s bonding information**	**O 1s bonding information**	**Mn 3s bonding information**	**Mn 2p bonding information**
C-C =284.79	Mn-O-Mn =529.73	$Mn3s_{3/2}$ =89.37	642.39
C-O =285.28	Mn-O-H = 531.53	$Mn3s_{1/2}$ =84.37	645.03
O-C=O =288.03	C-O = 533.42		653.99
Π-Π* =291.46	C=O = 535.09		657.57

Table S3. Comparison between energy density, life cycle, cell voltage, depth of discharge, cost, capital cost, efficiency, green and recyclability of Pb-acid and this Zn-MnO_2 battery.

Batteries types	Pb-acid	Ref.	Zn-MnO_2 (This work)
Energy density / Wh Kg^{-1}	35–50	[12,13]	673 $_{MnO2\ active\ materials}$
Life cycle	1000	[12,14]	400
Cell voltage / V	1.5-2	[13]	2.0
Depth of discharge (%)	30-50	[13-15]	10
Cost / US $ $(kW\ h)^{-1}$	-	-	~10*
Capital cost US $ $(kW\ h)^{-1}$	200-400	[10,12,14]	100-150
Efficiency (%)	65-85	[12,14]	~100
Recyclability (%)	90-95	[15]	>95
Green	No	[12,15]	Yes

*The calculation provided is based on estimations of raw material costs.[11, 16-18]

Table S4. Comparing the electrochemical performance of different configurations of cathodes, anodes, and electrolyte compositions as reported in relevant literature with the configuration of this Zn-MnO_2 battery.

Cathode	Anode	Electrolyte composition	Average cell voltage	Specific capacity	Performance/ capacity retention	Ref.
α-MnO_2	Zn metal foil	1 M $ZnSO_4$	1.3 V at 16 mA g^{-1}	323 mAh g^{-1} at 16 mA g^{-1}	46 % retained after 75 cycles at 83 mA g^{-1}	[19]
δ-MnO_2	Zn metal foil	1 M $ZnSO_4$	1.28 V at 83 mA g^{-1}	250 mAh g^{-1} at 83 mA g^{-1}	46 % retained after 100 cycles at 83 mA g^{-1}	[20]
δ-MnO_2	Zn metal foil	0.5M $Zn(CF_3SO_3)_2$ in acetonitrile	0.75 V at 12.3 mA g^{-1}	120 mAh g^{-1} at 12.3 mA g^{-1}	48 % retained after 125 cycles at 12.3 mA g^{-1}	[21]
γ-MnO_2	Zn metal foil	$Zn(CF_3SO_3)_2$ in PC+EC+PVDF	1.25 V at 200 μA cm^{-2}	105 mAh g^{-1} at 10 μA cm^{-2}	65 % retained after 65 cycles at 200 μA cm^{-2}	[22]
γ-MnO_2	Zn metal foil	1 M $ZnSO_4$	1.32 V at 0.05 mA cm^{-2}	285 mAh g^{-1} at 0.05 mA cm^{-2}	63 % retained after 40 cycles at 0.5 mA cm^{-2}	[23]
α-MnO_2	Zn metal foil	1 M $ZnSO_4$	1.26 V at 83 mA g^{-1}	353 mAh g^{-1} at 16 mA g^{-1}	63 % retained after 50 cycles at 83 mA g^{-1}	[24]
α-MnO_2	Zn metal foil	1 M $ZnSO_4$	1.3 V at 10.5 mA g^{-1}	195 mAh g^{-1} at 10.5 mA g^{-1}	70 % retained after 30 cycles at 42 mA g^{-1}	[25]
α-MnO_2@CNT	2 wt.% CMC, coated on zinc foil	2 M $ZnSO_4$ +0.5 M $MnSO_4$	1.35 V at 100 mA g^{-1}	665 mAh g^{-1} at 100 mA g^{-1}	99 % retained after 500 cycles at 5000 mA g^{-1}	[26]
MnO_x@ Ndoped C	Zn metal foil	2 M $ZnSO_4$ + 0.1 M $MnSO_4$	1.3 V at 100 mA g^{-1}	385 mAh g^{-1} at 100 mA g^{-1}	99 % retained after 1600 cycles at 2 A g^{-1}	[27]
MnO_2/rGO	Electropla	2 M $ZnSO_4$	1.37 V	332 mAh g^{-1}	96 % retained after	[28]

	ted Zn	+ 0.1 M $MnSO_4$	at 300 mA g^{-1}	at 300 mA g^{-1}	500 cycles at 6 A g^{-1}	
PANI-MnO_2	Zn metal foil	2 M $ZnSO_4$ + 0.1 M $MnSO_4$	1.36 V at 50 mA g^{-1}	298 mAh g^{-1} at 50 mA g^{-1}	89 % retained after 5000 cycles at 2 A g^{-1}	[29]
Electrolytic Zn-MnO_2	Zn foam	1 M $ZnSO_4$ + 1M $MnSO_4$ + 0.1 M H_2SO_4	1.95 V at 2 mA cm^{-2}	571 mAh g^{-1}	92 % retained after 1800 cycles at 30 mA cm^{-2}	[11]
Layered MnO_2	Zn metal foil	1 M $ZnSO_4$	1.3 V at 50 mA g^{-1}	289 mAh g^{-1} at 50 mA g^{-1}	35 % retained after 50 cycles at 100 mA g^{-1}	[30]
$ZnMn_2O_4$@PEDOT	Zn metal foil	1 M $ZnSO_4$	1.32 V at 80 mA g^{-1}	207 mAh g^{-1} at 80 mA g^{-1}	93.8 % retained after 300 cycles at 1290 mA g^{-1}	[31]
Spinel $ZnMn_2O_4$@C	Zn metal foil	3M $Zn(CF_3SO_3)_2$	1.36 V at 50 mA g^{-1}	150 mAh g^{-1} at 50 mA g^{-1}	94 % retained after 500 cycles at 500 mA g^{-1}	[32]
β-MnO_2	Zn metal foil	1 M $ZnSO_4$ +0.1 M $MnSO_4$	1.29 V at 100 mA g^{-1}	270 mAh g^{-1} at 100 mA g^{-1}	75 % retained after 200 cycles at 200 mA g^{-1}	[33]
**In situ grown β-MnO_2 on C-cloth**	**Zn metal foil**	**1 M $ZnSO_4$ +2 M $MnSO_4$**	**2 V at 2 mA cm^{-2}**	**571 mAh g^{-1} at 10 mA cm^{-2}**	**100 % retained after 400 cycles at 10 mA cm^{-2}**	**This work**

The specific capacity calculation of this Zn-MnO_2 battery in accordance with the two-electron MnO_2/Mn^{2+} reaction mechanism.

The calculation is based on the method described by Chao et al.[11]

The theoretical capacity of Zinc anode C_A = 820 mAh g^{-1}

The theoretical capacity of MnO_2 cathode C_C = 616 mAh g^{-1}

The theoretical energy density = 1/ (1/C_A + 1/ C_C) x E cell (2 V) = 704 Wh kg^{-1}

For this Zn-MnO_2 battery after 400 cycles the areal capacity = 2 mAh cm^{-2}

MnO_2 deposition 3.5 mg/cm^{-2}

Gravimetric capacity = 571 mAh g^{-1}

The energy density of this Zn-MnO_2 battery = 673 Wh kg^{-1}

Hence, the Gravimetric capacity calculated as ~571 mAh g^{-1} based on the two-electron mechanism and the mass of MnO_2 after deposition. The capacity analysis suggests that almost all of the deposited MnO_2 (~100%) was electrolyzed in the discharge process, which validates the proposed electrolysis/electrodeposition mechanism at the cathode.

The cost analysis calculation of this Zn-MnO_2 battery.

The cost analysis relies on the estimations of raw material costs.[11,16-18]

The price of MnO_2 is 4.51 US$ kg^{-1}

The price of zinc is 2.725 US$ kg^{-1}

$ZnSO_4$, $MnSO_4$ nearly 0.1 US$ kg^{-1}

Zinc mass fraction in $Zn-MnO_2$ full cell:

Capacity of MnO_2/Capacity of (MnO_2 + Zinc) = 0.4105

Therefore, mass of zinc for 1 Kg of cell = 410.5 g of zinc

MnO_2 mass fraction in $Zn-MnO_2$ full cell:

Capacity of zinc/Capacity of (Zinc + MnO_2) = 0.5895

Therefore, mass of MnO_2 for 1 Kg of cell = 589.5 g of MnO_2

Cost of active materials of 1Kg cell = 3.778 US$ kg^{-1}

Therefore, cost of energy storage = 5.614 US$ $(kW\ h)^{-1}$

(This cost is based on the active material mass only, cost of other than active material excluded)

While the inclusion of supplementary cell components and housing is expected to elevate the overall cost of the battery, the manufacturing cost for this $Zn-MnO_2$ battery are projected to cap at approximately 10 US$ per kWh. This cost arises from the facile fabrication process conducted at ambient atmospheric conditions, devoid of complicated procedures or extra protections. Moreover, the estimated capital cost for this $Zn-MnO_2$ battery ranges between 100-150 US$ per kW h.

References

[1] H. U. Shah, F. Wang, M. S. Javed, M. A. Ahmad, M. Saleem, J. Zhan, Z. U. H. Khan, Y. Lia, *J. of Energy Storage* **2018**, *17*, 318–326.

[2] F. Buciuman, F. Patcas, R. Craciun, D. R. T. Zahn, *Phys. Chem. Chem. Phys*. **1999**, *1*, 185–190.

[3] A. M. Toufiq, F. Wang, Q. Javed, Y. Li, *Nanotechnology* **2013**, *24*, 415703.

[4] M. R. Panda, A. R. Kathribail, B. Modak, S. Sau, D. P. Dutta, S. Mitra, *Electrochim. Acta* **2021**, *392*, 139026.

[5] A. Raj. K, M. R. Panda, D. P. Dutta, S. Mitra, *Carbon* **2019**, *143*, 402–412.

[6] G. Li, W. Chen, H. Zhang, Y. Gong, F. Shi, J. Wang, R. Zhang, G. Chen, Y. Jin, T. Wu, Z. Tang, and Y. Cui, *Adv. Energy Mater*. **2020**, *10*, 1902085.

[7] L. Zhang, L. Tu, Y. Liang, Q. Chen, Z. Li, C. Li, Z. Wanga and W. Li, *RSC Adv*. **2018**, *8*, 42280.

[8] R. A. Davoglio, G. Cabello, J. F. Marco, S. R. Biaggio, *Electrochim. Acta* **2018**, *261*, 428–435.

[9] A. R. Hsu, H. -H. Chien, C. -Y. Liao, C. -C. Lee, J. -H. Tsai, C. -C. Hsu, I. -C. Cheng, J. -Z. Chen, *Coatings* **2018**, *8*, 52.

[10] C. Xie, Y. Duan, W. Xu, H. Zhang, and X. Li, *Angew. Chem. Int. Ed*. **2017**, *56*, 14953–14957.

[11] D. Chao, W. Zhou, C. Ye, Q. Zhang, Y. Chen, L. Gu, K. Davey, and S. -Z. Qiao, *Angew. Chem. Int. Ed*. **2019**, *58*, 7823–7828.

[12] F. Rahman, S. Rehman, M. A. Abdul-Majeed, *Renew. Sustain. Energy Rev*. **2012**, *16*, 274–283.

[13] Z. Yang, J. Zhang, M. C. Kintner-Meyer, X. Lu, D. Choi, J. P. Lemmon, J. Liu, *Chem. Rev*. **2011**, *111*, 3577–3613.

[14] N. D. Ingale, J. W. Gallaway, M. Nyce, A. Couzis, S. Banerjee, *J. Power Sources* **2015**, *276*, 7–18.

[15] K. Yanamandra, D. Pinisetty, A. Daoud, N. Gupta, *J. Indian Inst. Sci*. **2022** 102(1), 281–295.

[16] https://minerals.usgs.gov/minerals/pubs/commodity/ Nov 2023.

[17] Costs are from USGS Materials Databook.

[18] J-Carbon Materials Co., Ltd. National Renewable Energy Laboratory Technical Report *NREL/TP-7A40-73238* June **2019**.

[19] M. H. Alfaruqi, S. Islam, J. Gim, J. Song, S. Kim, D. T. Pham, J. Jo, Z. Xiu, V. Mathew, J. Kim, *Chem Phys Lett* **2016**, *650*, 64–68.

[20] M. H. Alfaruqi, J. Gim, S. Kim, J. Song, D. T. Pham, J. Jo, Z. Xiu, V. Mathew, J. Kim, *Electrochem. commun.* **2015**, *60*, 121–125.

[21] S. -D. Han, S. Kim, D. Li, V. Petkov, H. D. Yoo, P. J. Phillips, H. Wang, J. J. Kim, K. L. More, B. Key, R. F. Klie, J. Cabana, V. R. Stamenkovic, T. T. Fister, N. M. Markovic, A. K. Burrell, S. Tepavcevic, J. T. Vaughey, *Chem Mater* **2017**, *29*, 4874–4884.

[22] G. Kumar, *Solid State Ion.* **2003**, *160*, 289–300.

[23] M. H. Alfaruqi, V. Mathew, J. Gim, S. Kim, J. Song, J. P. Baboo, S. H. Choi, J. Kim, *Chem Mater.* **2015**, *27*, 3609–3620.

[24] M. H. Alfaruqi, J. Gim, S. Kim, J. Song, J. Jo, S. Kim, V. Mathew, J. Kim, *J. Power Sources* **2015**, *288*, 320–327.

[25] B. Lee, H. R. Lee, H. Kim, K. Y. Chung, B. W. Cho, S. H. Oh, *Chem. Commun.* **2015**, *51*, 9265–9268.

[26] D. Xu, B. Li, C. Wei, Y. -B. He, H. Du, X. Chu, X. Qin, Q. -H. Yang, F. Kang, *Electrochim. Acta* **2014**, *133*, 254–261.

[27] Y. Fu, Y. Fu, Q. Wei, G. Zhang, X. Wang, J. Zhang, Y. Hu, D. Wang, L. Zuin, T. Zhou, Y. Wu, S. Sun, *Adv. Energy Mater.* **2018**, *8*, 1801445.

[28] Y. Huang, J. Liu, Q. Huang, Z. Zheng, P. Hiralal, F. Zheng, D. Ozgit, S. Su, S. Chen, P. -H. Tan, S. Zhang, H. Zhou, *npj flex. electron.* **2018,** *2*, 21.

[29] J. Huang, Z. Wang, M. Hou, X. Dong, Y. Liu, Y. Wang, Y. Xia, *Nat. Commun.* **2018**, *9*, 2906.

[30] M. H. Alfaruqi, S. Islam, D. Y. Putro, V. Mathew, S. Kim, J. Jo, S. Kim, Y. -K. Sun, K. Kim, J. Kim, *Electrochim. Acta* **2018**, *276*, 1–11.

[31] H. Zhang, J. Wang, Q. Liu, W. He, Z. Lai, X. Zhang, M. Yu, Y. Tong, X. Lu, *Energy Storage Mater.* **2019**, *21*, 154–161.

[32] N. Zhang, F. Cheng, Y. Liu, Q. Zhao, K. Lei, C. Chen, X. Liu, J. Chen, *J. Am. Chem. Soc.* **2016**, *138*, 12894–12901.

[33] S. Islam, M. H. Alfaruqi, V. Mathew, J. Song, S. Kim, S. Kim, J. Jo, J. P. Baboo, D. T. Pham, D. Y. Putro, Y. -K. Sunb, J. Kim, *J. Mater. Chem. A* **2017**, *5*, 23299–23309.
